# A novel synthetic melanin as a potential anticancer agent that induces apoptosis and cyclin D downregulation through distinct pathways

**DOI:** 10.1016/j.jbc.2026.113065

**Published:** 2026-04-24

**Authors:** Yoshiyuki Kawamoto, Yui Furuhashi, Silvi Zakiyatul Ilmiyah, Seiji Yamaguchi, Nozomi Nishimura, Riko Iwata, Arisa Imoto, Takashi Murate, Yuhsuke Ohmi, Sofy Permana, Agustina Tri Endharti, Yuki Ueno, Machiko Iida, Ichiro Yajima, Nobutaka Ohgami, Masashi Kato, Kozue Takeda

**Affiliations:** 1 Department of Biomedical Sciences, College of Life and Health Sciences, Chubu University, Kasugai, Aichi, Japan; 2 Doctoral Program in Medical Sciences, Faculty of Medicine, Universitas Brawijaya, Malang, Indonesia; 3 Department of Clinical Engineering, College of Life and Health Sciences, Chubu University, Kasugai, Aichi, Japan; 4 Department of Biology, Faculty of Mathematics and Natural Sciences, Universitas Brawijaya, Malang, Indonesia; 5 Department of Clinical Parasitology, Faculty of Medicine, Universitas Brawijaya, Malang, Indonesia; 6 Department of Health and Nutritional Sciences, Faculty of Health Sciences, Aichi Gakuin University, Nisshin, Aichi, Japan; 7 Department of Disease Model, Institute For Developmental Research, Aichi Developmental Disability Center, Kasugai, Aichi, Japan; 8 Unit of Molecular and Cellular Toxicology, Department of Bioscience and Engineering, College of Systems Engineering and Science, Shibaura Institute of Technology, Saitama, Japan; 9 Department of Hygiene, Fujita Heath University School of Medicine, Toyoake, Aichi, Japan; 10 Department of Occupational and Environmental Health, Nagoya University Graduate School of Medicine, Nagoya, Aichi, Japan

**Keywords:** anticancer drug, apoptosis, calpain, cyclin D, proteolysis

## Abstract

Despite extensive development of anticancer agents, there remains a critical need for therapeutics with novel mechanisms of action. This study reports the potent anticancer activity and mechanistic analysis of highly water-soluble dopa-melanin (DM) prepared through a unique synthetic process. DM exhibits distinctive structural and chemical properties that contribute to its exceptional aqueous solubility (≥50 mg/ml) and may underlie its enhanced biological activity. DM reduced cell viability in multiple cancer cell lines. Using HeLa cells as a representative model, DM suppressed cell migration and three-dimensional growth. Fractionation revealed that activity was associated with polymers larger than 30 kDa, suggesting a critical role for high-molecular-weight species. Mechanistic studies showed that DM induced S/G_2_/M arrest followed by cell death with minimal apoptotic body formation. DM rapidly decreased cyclin D1 and D3 protein and mRNA levels. A pan-caspase inhibitor significantly suppressed DM's inhibitory effect on cell viability but did not prevent cyclin D degradation. Cyclin D degradation was mediated through calcium-dependent calpain activation triggered by endoplasmic reticulum Ca^2+^ release *via* IP_3_ receptors, and inhibition of this pathway attenuated DM's activity. In a mouse syngeneic tumor model, oral administration of DM significantly inhibited tumor growth without apparent toxicity. These results demonstrate that both caspase-dependent apoptosis and a caspase-independent cyclin D degradation pathway contribute to DM-induced cell death. Its high solubility, oral efficacy, and unique mechanism of action make DM a promising candidate for therapeutic development.

Melanin is a naturally occurring pigment widely distributed across various organisms. In animals, it is synthesized and polymerized within specialized pigment cells called melanocytes. The biosynthesis of melanin originates from the amino acid tyrosine, which, under the action of the enzyme tyrosinase, leads to the formation of eumelanin in the absence of cysteine or pheomelanin in its presence. In the skin, exposure to ultraviolet (UV) radiation activates melanocytes, prompting the synthesis of melanin, which is then released extracellularly in the form of melanosomes and transferred to keratinocytes where the melanin becomes visible. The melanin retained in keratinocytes absorbs UV radiation, thereby protecting deeper cells from UV-induced damage (1, 2). Additionally, melanocytes are present in the choroid and retinal pigment epithelium of the eye, where they contribute to the regulation of light entering the eye. Melanocytes are also found in the inner ear (3), although their role in this location remains largely unclear. To date, natural melanin has been reported to possess anti-inflammatory (4, 5, 6), antioxidant (7, 8, 9), and antivenin (10) properties, suggesting potential applications in medical treatments.

Melanin secreted by melanocytes is typically eliminated from the body through cellular turnover mechanisms. Despite a thorough understanding of the melanin biosynthetic pathway and the feasibility of its industrial chemical synthesis, the application of either synthetic or naturally extracted melanin in the life sciences and medical fields remains limited. To our knowledge, few studies have investigated the artificial modulation of melanin activity through its application to cultured cells or animal models, particularly regarding its effects on cell viability. This limited research may be attributed to the inherent challenges associated with melanin’s physicochemical properties, such as its heterogeneous polymeric nature and poor solubility in various solvents, which complicates the experimental assessment of its biological activity.

Conventionally, melanin exhibits poor solubility in most hydrophobic and hydrophilic solvents under simple stirring conditions. However, our previous work demonstrated that commercially available melanin can be readily solubilized up to 50 mg/ml in 500 mM HEPES (2-[4-(2-hydroxyethyl)-1-piperazinyl]) ethanesulfonic acid), pH 7.5, a physiological buffer solution. We reported that this synthetic melanin preparation strongly inhibits antigen-induced degranulation in rat basophilic leukemia cells (RBL-2H3) (11).

Over the course of this investigation, we preliminarily observed that commercial melanin dissolved in HEPES strongly suppressed the viability of RBL-2H3 cells. Previous studies have demonstrated that plant-derived melanin (allomelanin, herbal melanin) inhibits the proliferation of cells derived from human intestinal carcinoma and colorectal cancer (12, 13, 14). However, reports on the direct inhibitory effects of synthetic melanin on cancer cells remain limited. In this study, we report the successful synthesis and modification of high-purity water-soluble melanin with a defined molecular weight range and unique structural characteristics, distinct from those previously reported. We demonstrate that this novel melanin preparation exhibits potent inhibitory effects on cancer cell viability and explore the underlying molecular mechanisms. We conducted a comprehensive structural analysis of the synthetic melanin and hypothesis-driven investigations into its *in vitro* inhibitory effects on the viability of cultured cancer cells. Additionally, we performed validation studies in animal models. Based on these findings, we propose that this novel melanin formulation has potential as a promising candidate for anticancer therapy.

## Results

### Comparison of characteristics of water-soluble melanin and commercial products

We produced water-soluble melanin with a molecular weight adjusted to below a specified threshold. This synthetic melanin was produced through oxidative polymerization using only sodium hydroxide, with L-dopa as the starting material. Subsequently, it was neutralized with hydrochloric acid, desalted using a dialysis membrane with a molecular weight cutoff of 20,000, and then lyophilized to obtain a powdered form. The molecular weight distribution of this synthetic compound was examined using size-exclusion chromatography. As shown in Table 1, approximately 95% of the molecules had a molecular weight of 10,000 or less. The modal distribution revealed that around 49% were distributed within the range of 3000 to 10,000. However, it appears that the compound is a mixture of various molecular weights. The number-average molecular weight (M_n_) and weight-average molecular weight (M_w_) are 1400 and 4900, respectively, with a calculated polydispersity index (PDI) of 3.5 (Table 2).

**Table 1:** Molecular weight distribution of DM

Molecular weight range	Peak area (%)
700,000 ∼	0
300,000 ∼ 700,000	0
100,000 ∼ 300,000	Faint
30,000 ∼ 100,000	1
10,000 ∼ 30,000	4
3000 ∼ 10,000	49
1000 ∼ 3000	27
∼ 1000	19
Total	100

**Table 2:** Number average molecular weight (Mn), weight average molecular weight (Mw), and polydispersity index (PDI) of DM

Mn	Mw	PDI (Mn/Mw)
140	4900	3.5

**Figure 1:**
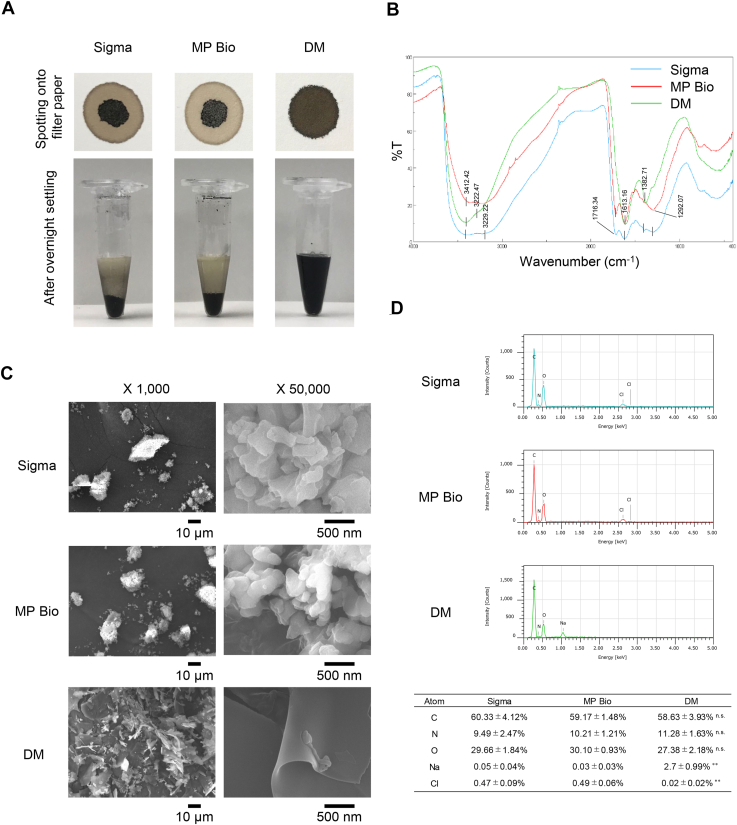
**Physicochemical characterization and structural comparison of commercial melanins and DM.***A*, Comparison of water solubility among commercially available melanin (Sigma-Aldrich and MP-Biomedicals) and DM when added to ultrapure water. Each powder sample was added to ultrapure water at a concentration of 50 mg/ml and thoroughly mixed. *Upper panel*: 20 μl of each solution was spotted on filter paper immediately after mixing. *Lower panel*: The same solutions after 1 week of static incubation. *B*, FT-IR spectra of the various melanin samples. The numbers indicate wavenumbers (cm^-1^). *C*. Scanning electron microscopy (SEM) images of the various melanin samples. Images are shown at 1000 × and 500,00 × magnification. *D*, Elemental analysis of various melanin samples using scanning microscopy with energy dispersive X-ray spectroscopy (SEM-EDX). Five locations were randomly selected from each SEM image for elemental analysis. A representative image is shown. The proportions of each element are presented in the table below. Data are presented as mean ± SD (n = 5 per group). Statistical analysis was performed using one-way ANOVA followed by the Tukey–Kramer *post hoc* test. For Na content, one-way ANOVA yielded F(2, 12) = 35.84, *p*= 8.7 × 10^-6^; for Cl content, F(2, 12) = 86.15, *p*= 7.62 × 10^-8^. Sigma melanin and MP-Bio melanin were compared with the DM group. ∗∗*p*< 0.01, n.s.: not significant.

We conducted an analysis comparing the post-solubilization homogeneity and molecular structure of the DM produced with commercially available synthetic melanin products (Sigma-Aldrich [Bulington, MA] and MP-Biomedicals). Initially, we investigated homogeneity after dissolving in distilled water. We adjusted each melanin to a concentration of 50 mg/ml, vigorously mixed the solutions, and spotted them onto filter paper. As a result, the two commercial products formed spots with a central dark area and a lighter periphery, exhibiting variations in intensity, while DM formed a uniform spot. Furthermore, after allowing sufficient settling overnight, sedimentation was evident in the commercially available products, whereas in DM, there was minimal observable separation between water and suspended particles (Fig. 1*A*).

Next, we performed Fourier transform infrared (FTIR) spectroscopy analysis to investigate the functional groups and to compare the structural similarities of each melanin product (Fig. 1*B*). The results showed that the two commercial melanins exhibited nearly identical absorption spectra, while DM displayed a slightly different absorption spectrum compared to commercial ones. All materials exhibited a broad absorption band at 3680–2200 cm^-1^ and an absorption band at 1613 to 1618 cm^-1^. The former was attributed to the stretching vibrations (O-H and N-H) of amino groups, amido groups, carboxyl groups, phenol groups, and aromatic amino groups present in the indole and pyrrole systems. The latter was assigned to the aromatic C=C stretching and the COO-symmetric stretching. A peak at approximately 1717 cm^-1^ was observed in the commercial samples but not in DM. This band suggests the presence of C=O stretching in ketones, aryl ketones, and carboxylic acids. Additionally, in the fingerprint region below 1500 cm^-1^, an absorption band was observed around 1290 cm^-1^, especially in the commercial samples, but was significantly reduced in DM. This band suggests the presence of C=O stretching in esters and carboxylic acids. These results suggest that DM has structural differences compared to the commercial melanins.

Next, we investigated the morphology of each synthesized melanin using scanning electron microscopy (SEM). The results revealed distinct differences in surface structure between the two commercial melanin and DM samples. The former exhibited a lumpy morphology characterized by agglomerated and non-uniform aggregates, while the latter had a smooth surface with a smaller thickness (Fig. 1*C*). In parallel with SEM observations, we conducted elemental analysis using energy dispersive X-ray spectroscopy (EDX). Intriguingly, Cl was detected in addition to C, N, and O in commercial melanin but not in DM, while Na was detected in DM but not in commercial melanin (Fig. 1*D*). Based on these results, it can be concluded that the DM we independently synthesized exhibits unique properties in terms of both morphology and physical properties, which are different from those of at least two commercially available synthetic melanins.

### DM suppresses cancer cell viability and growth

**Figure 2:**
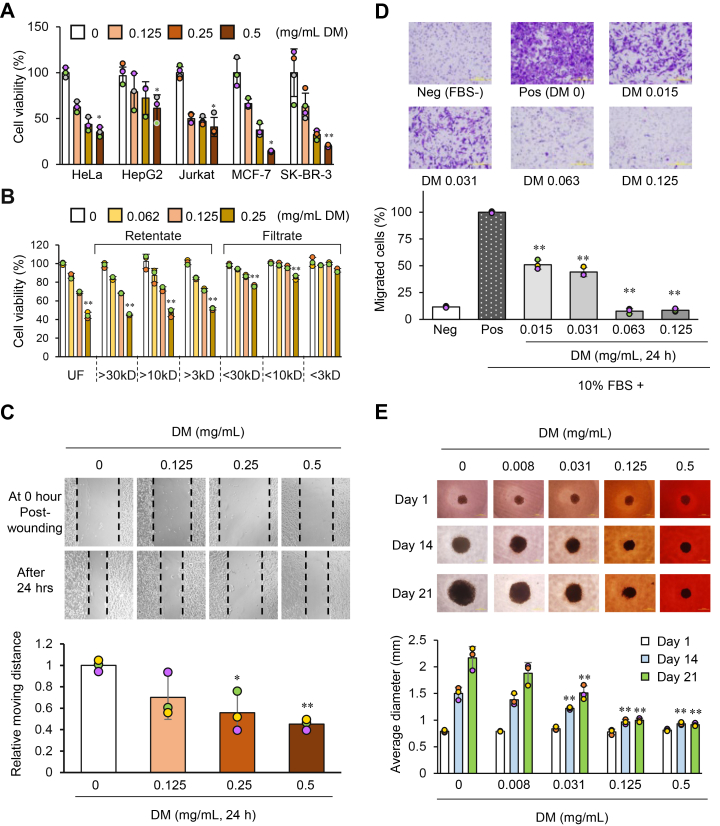
**Effects of DM on cancer cell viability and growth.***A*, Cell viability assay. Various cultured cancer cell lines were seeded into culture plates (1 × 10^4^cells) and immediately treated with different concentrations of DM. After 24 h, viable cells were quantified using the method described in the Experimental procedures section. The graph shows relative cell viability compared to the control (0 mg/ml DM, water). *B*, cell viability assay using molecular weight–fractionated DM. HeLa cells (1 × 10^4^cells) were seeded into 96-well plates and treated with various concentrations of molecular weight–fractionated DM as described in the Experimental procedures section. After 48 h, viable cells were quantified according to the method described in the Experimental procedures section. The graphs show the relative cell viability compared with the control (0 mg/ml DM, water). UF denotes the unfractionated sample. Statistical analysis was performed on three to four biological replicates using the Kruskal–Wallis test followed by Bonferroni correction and Dunn’s test for multiple comparisons. Statistical significance was determined relative to control (DM 0 mg/ml). ∗*p*< 0.05, ∗∗*p*< 0.01. *C*, Wound-healing assay. Confluent HeLa cells were wounded and then treated with various concentrations of DM for 24 h. The relative migration distance compared to the control (0 mg/ml DM, water) was calculated using image analysis and is presented in the graph. Statistical analysis was performed on three biological replicates using one-way ANOVA (F(3, 8) = 8.252, *p*= 0.00785) followed by Dunnett’s test, comparing each treatment group to the control (0 mg/ml DM) to determine statistical significance (∗*p*< 0.05, ∗∗*p*< 0.01). *D*, migration assay. HeLa cells were seeded into Boyden chambers, and various concentrations of DM were added to the upper chamber. After 24 h, the cells that had migrated through the membrane pores were stained with crystal violet, and the number of cells in a defined area was quantified. The graph shows the relative cell number compared to the positive control (without DM treatment). Statistical analysis was performed on three biological replicates using one-way ANOVA (F(4, 10) = 475.4, *p*= 2.34 × 10^-11^) followed by Dunnett’s test, comparing each treatment group to the positive control (Pos) to determine statistical significance (∗∗*p*< 0.01). Neg: Negative control (10% FBS-, DM-); Pos: Positive control (10% FBS+, DM-). *E*. Effect of DM on long-term cultured cancer cells in a 3D culture. HeLa cells were seeded into specialized 3D culture plates and simultaneously treated with various concentrations of DM. Optical microscopy images were captured at 1, 14, and 21 days after DM addition. Representative images for each concentration and time point are shown. Spheroid diameters were measured using image analysis software and are presented in the graph. Statistical analysis was performed on three biological replicates using one-way ANOVA followed by Dunnett’s test, comparing each treatment group to the control (0 mg/ml DM, water) for each day to determine statistical significance. Day 1: F(4, 10) = 1.647, *p*= 0.238; Day 14: F(4, 10) = 32.62, *p*= 1.03 × 10^-5^; Day 21: F(4, 10) = 41.96, *p*= 3.22 × 10^-6^. ∗∗*p*< 0.01; n.s., not significant.

As described in the Introduction section, we observed a potential decrease in the viability of RBL-2H3, a rat cell line derived from basophilic leukemia cells, treated with DM. To further investigate this phenomenon, we conducted cell viability assays using a panel of human-derived cancer cell lines. The cell lines used were HeLa (cervical cancer), HepG2 (hepatoblastoma), Jurkat (T-cell leukemia), MCF-7 (Her2-negative breast adenocarcinoma), and SK-BR-3 (Her2-positive breast adenocarcinoma). In the cell viability assay, each cultured cell was seeded at a low density, and cell viability was measured 24 h after the addition of DM. As a result, DM-dependent reduction in cell viability was observed in all examined cancer cell lines (Fig. 2*A*).

We next examined whether commercially available synthetic melanin exhibits similar inhibitory effects on cell viability. Due to the extremely poor solubility of commercial melanin in neutral solutions and organic solvents, we prepared the melanin solutions by sonication using an ultrasonic homogenizer. The sonicated synthetic melanin solution demonstrated cancer cell growth inhibition comparable to that observed with DM (Fig. S1).

These results suggest that both DM and commercial melanin possess common inhibitory properties against cancer cell viability. The commercial melanin samples also reduced cell viability and showed stronger inhibitory effects than did DM. However, the particle size was not uniform, making it difficult to administer the compound at an accurate concentration, and larger particles often caused cells to adhere and to be lost during washing. In addition, the preparation of commercial melanin required specialized equipment and was labor intensive and costly.

In contrast, DM formed a stable and homogeneous solution that could be prepared easily and inexpensively. Thus, DM was used in subsequent experiments. Based on these findings and the expectation that most cancer cell lines would exhibit similar growth inhibitory responses, we selected HeLa cells as a representative cancer cell line and DM as the melanin sample for subsequent experiments.

As previously noted (Tables 1 and 2), DM is a mixture of polymers with various chain lengths. Could this heterogeneity influence the inhibitory effects on cancer cells viability? To address this question, we fractionated synthetic DM into several molecular weight ranges using ultrafiltration membranes with different pore sizes and then compared the inhibitory effects on cell viability per unit concentration of DM in each fraction (Fig. 2*B*). Ultrafiltration membranes with molecular weight cut-off (MWCO) values of 30 kDa, 10 kDa, and 3 kDa were used for the molecular weight fractionation. After processing with each membrane, the retentate (material retained in the filter) and the filtrate were collected, adjusted to the appropriate stock concentrations, and applied to HeLa cells at various concentrations for 48 h, followed by the measurement of cell viability. In all cases, the retentates exhibited concentration-dependent growth-inhibitory effects on par with those of unfractionated (UF) DM. This finding suggests that polymers with molecular weights of ≥30 kDa possess sufficient inhibitory effects on cell viability.

In contrast, the smaller the molecular weight of the filtrate, the weaker the growth-inhibitory effect of DM. These results indicate that the efficacy of DM polymers in inhibiting cell viability increases with molecular weight, with components of ≥30 kDa exhibiting substantial activity, whereas the contribution of lower molecular weight species is limited. As the ≥30 kDa fraction accounts for the primary activity, unfractionated DM containing all molecular weight components was deemed sufficient to achieve the maximal effect. Therefore, unfractionated DM was used in subsequent experiments.

To evaluate the effects of DM on cancer cell migration, we performed a wound-healing assay and a transwell migration assay. As expected, DM inhibited the rate of wound closure (Fig. 2*C*) and reduced the number of cells that migrated through the porous membrane in a dose-dependent manner (Fig. 2*D*). It should be noted that these assays were conducted at 24 h, at which point cell migration, proliferation, and cell survival may all contribute to the observed results.

Having evaluated the effects of DM in monolayer cultures, we further investigated its effects using a 3D spheroid culture model, which better mimics the morphology of solid tumors. HeLa cells were cultured with various concentrations of DM for up to 3 weeks, and spheroid size was assessed over time. As shown in Figure. 2*E*, DM suppressed the increase in spheroid size in a dose-dependent manner. At 0.5 mg/ml, spheroid growth was markedly reduced, with little increase in diameter observed between days 14 and 21. In contrast, spheroids in the control group continued to increase in size over time.

Based on these findings, DM reduces the viability of human-derived cancer cell lines and suppresses tumor cell expansion under both 2D and 3D culture conditions.

### Cell cycle analysis using Fucci-transduced HeLa cells

**Figure 3:**
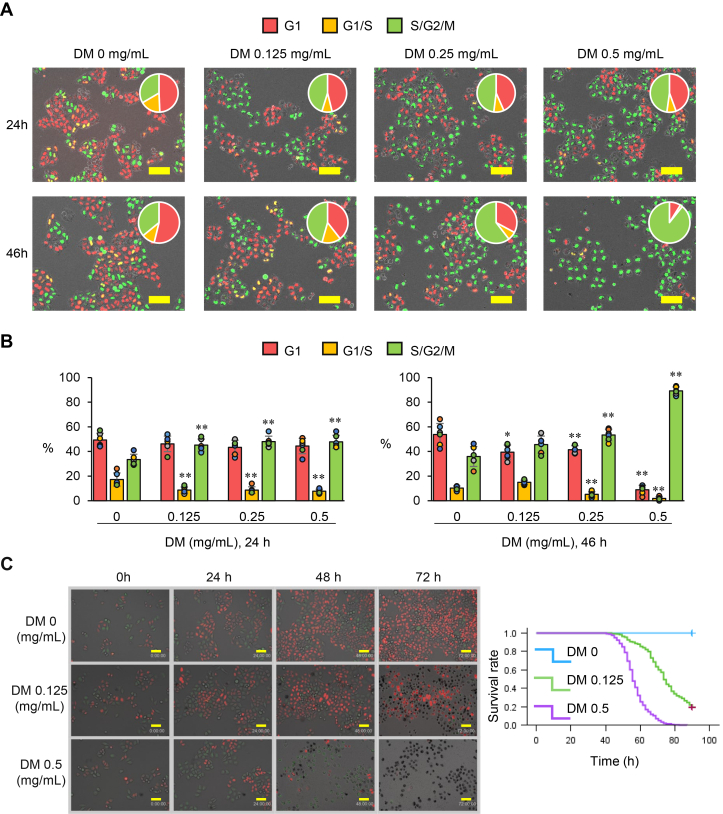
**Cell cycle analysis and cell death induction observation using HeLa/Fucci cells**. *A*, representative fluorescence microscopy images. Cells were treated with various concentrations of DM and observed under a fluorescence microscope at 24 h and 46 h post-treatment. The number of cells in the G1, G1/S, and S/G2/M phases within a defined area was quantified, and their respective proportions are displayed in pie charts. Scale bar = 100 μm. *B*, Proportions of cells in each cell cycle phase at 24 h and 46 h post-DM treatment. Statistical analysis was performed on six biological replicates using Dunnett’s test, comparing the proportion of cells in each phase of each treatment group to the control (0 mg/ml DM, water) to determine statistical significance (∗*p*< 0.05, ∗∗*p*< 0.01). *C*, long-term observation of cells following DM treatment. *Left panel*: Representative images of cells up to 72 h post-DM treatment. *Right panel*: Cell survival curve up to 90 h post-DM administration. Time-lapse images were acquired every 90 min across three independent fields of view. At each time point, the total number of cells and the number of dead cells were counted per field of view. Dead cells were readily identified by their characteristically dark appearance and cessation of movement. The percentage of viable cells was calculated relative to the total cell count and plotted over time. Scale bar = 100 μm.

To elucidate the mechanisms of cell growth inhibition by DM treatment, we investigated which phase of the cell cycle was affected. Fucci (a fluorescent ubiquitination-based cell cycle indicator) is a tool for visualizing the cell cycle (15). To investigate the effects of DM treatment on the cell cycle, we utilized HeLa cells (HeLa/Fucci) that exhibit red fluorescence in the G1 phase, yellow fluorescence in the G1/S transition phase, and green fluorescence in the S/G2/M phase to obtain fluorescence images. HeLa/Fucci cells were treated with various concentrations of DM, and fluorescent observation images of the cells were acquired at 24 and 46 h. The percentages of cells in the G1 phase, G1/S transition phase, and S/G2/M phase were measured (Fig. 3, *A* and *B*). At 24 h after DM treatment, the percentage of G1-phase cells remained constant in all concentrations. However, compared to the control, the percentage of cells in the G1/S transition phase was significantly decreased, while the percentage of cells in the S/G2/M phase was significantly increased. At this treatment time, no concentration-dependent effect of DM treatment was observed. Interestingly, at 46 h post-DM treatment, there was a concentration-dependent decrease in the proportion of cells in the G1 and G1/S phases, which was inversely correlated with an increase in the proportion of cells in the S/G2/M phase. These results suggest that exposure to DM leads to G1/S transition followed by S/G2/M arrest in a majority of the cells.

After 46 h of treatment with DM, what is the fate of HeLa cells? To address this question, we extended our observations beyond 46 h. In the case of 0.125 mg/ml DM, the number of dead cells gradually increased around 40 h after treatment, and approximately half of the cells died after 72 h. When treated with 0.5 mg/ml DM, all cells died within 96 h (Figs. 3*C* and S2*A*).

It is desirable for anticancer agents to exhibit low toxicity towards normal cells. To compare the differential sensitivity to DM between HeLa cells and normal cells, we employed the normal human keratinocyte-derived cell line HaCaT and normal human fibroblast WI-38 cells. These cell lines, along with HeLa cells, were exposed to DM at a concentration of 0.25 mg/ml. Time-lapse observations were conducted for up to 72 h to monitor cellular behavior. The results revealed that both HaCaT and WI-38 cells maintained their motility and proliferative capacity throughout the observation period. In contrast, a significant decrease in viability was observed in HeLa cells after 72 h of exposure to DM. These findings strongly suggest that DM exhibits markedly lower cytotoxicity toward normal cells compared to HeLa cells (Fig. S2*B*).

These results provide compelling evidence that the synthesized melanin possesses high potential as a promising anticancer agent. In this study, we aimed to elucidate the detailed molecular mechanisms of DM-induced cell cycle arrest, which ultimately leads to cell death. To this end, we conducted the following experiments.

### Expression analysis of cell cycle regulatory proteins

In contrast to normal cells, cancer cells exhibit deregulated cell cycle control, leading to uncontrolled repetition of cell division. Cell cycle progression requires the presence of cyclin and cyclin-dependent kinase (CDK) complexes, and specific factors that function in each of the four phases (G1, S, G2, and M) of the cell cycle have been identified, and their roles elucidated in detail (14). During the G1/S transition, D-type cyclins (D1, D2, and D3) form complexes with CDK4 and CDK6, promoting the expression of E-type cyclins (E1, E2). Cyclin E forms a complex with CDK2, facilitating the activation of its transcriptional regulator Rb and contributing to S-phase entry.

**Figure 4:**
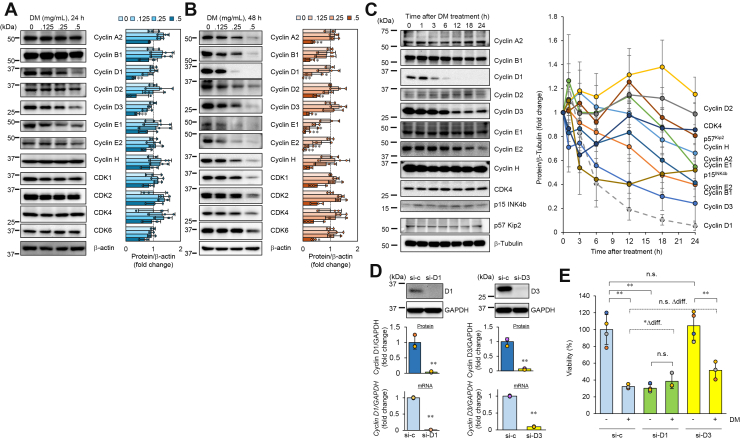
**Protein expression analysis of cell cycle regulatory factors**. *A* and *B*, effect of DM concentration on protein expression of cell cycle regulatory factors. HeLa cells were treated with various concentrations of DM for 24 h (*A*) or 48 h (*B*), and protein levels of the cyclins and CDKs shown in the figure were detected. β-actin was used as a normalizer. Statistical analysis was performed on three biological replicates using one-way ANOVA followed by Dunnett’s test, comparing each treatment group to the control (0 mg/ml DM, water) to determine statistical significance. ANOVA results were as follows — 24 h: Cyclin D1, F(3, 8) = 20.82, *p*= 3.9 × 10^-4^; Cyclin D3, F(3, 8) = 15.01, *p*= 1.19 × 10^-3^. 48 h: Cyclin A2, F(3, 8) = 19.2, *p*= 5.17 × 10^-4^; Cyclin B1, F(3, 8) = 5.094, *p*= 0.0292; Cyclin D1, F(3, 8) = 17.66, *p*= 6.9 × 10^-4^; Cyclin D2, F(3, 8) = 10.29, *p*= 4.04 × 10^-3^; Cyclin D3, F(3, 8) = 10.89, *p*= 3.38 × 10^-3^; Cyclin E1, F(3, 8) = 8.066, *p*= 8.39 × 10^-3^; Cyclin E2, F(3, 8) = 16.2, *p*= 9.25 × 10^-4^; CDK6, F(3, 8) = 11.23, *p*= 3.07 × 10^-3^. ∗*p*< 0.05, ∗∗*p*< 0.01. *C*, time-dependent effect of DM treatment on protein expression of cell cycle regulatory factors. HeLa cells were treated with 0.5 mg/ml DM for various durations, and the protein levels of the cyclins, CDKs, and CKIs shown in the figure were detected. β-tubulin was used as a normalizer. Band intensities obtained from Western blotting were quantified using the method described in the Experimental procedures section. The relative expression levels of each protein compared to the untreated control are graphed. These experiments were independently repeated at least twice, and representative data are shown. *D*, validation of cyclin D1 and cyclin D3 knockdown by RT-qPCR. HeLa cells were transfected with siRNA targeting cyclin D1 or cyclin D3. Protein expression levels were analyzed by Western blotting (*upper panel*), and gene expression levels were analyzed by RT-qPCR (*lower panel*). Expression values were normalized to GAPDH as an internal reference gene. Data are presented as mean ± SD (n = 3–4). Statistical significance was assessed using Welch’s *t* test (∗∗<0.01). *E*, effect of cyclin D1 or cyclin D3 knockdown on DM-induced suppression of HeLa cell viability. HeLa cells were transfected with control siRNA, cyclin D1 siRNA, or cyclin D3 siRNA using the reverse transfection method. Twenty-four hours after transfection, the cells were treated with DM at 0.25 mg/ml for 48 h. Cell viability was measured after treatment and expressed as the mean ± SD (n = 3–4). Within-group comparisons (No DM *versus* DM+) were analyzed using Welch’s *t* test (one-sided), with significance indicated as ∗*p*< 0.05, ∗∗*p*< 0.01, or “n.s.” (not significant). Among-group comparisons under the No DM condition (control siRNA, cyclin D1 siRNA, cyclin D3 siRNA) were performed using Welch’s *t* test (two-sided). The difference in the magnitude of the DM effect between the control and knockdown groups (Δdiff.) was tested using a one-sided permutation test; corresponding *p*-values or “n.s.” are shown.

In the late S phase, CDK2 is activated by cyclin A2 (cyclin A1 is found in germ cells), promoting the transition from the S to G2 phase. Subsequently, CDK1 forms a complex with cyclin A2, becoming activated and driving entry into the M phase. During the M phase, the CDK1-cyclin B complex contributes to the regulation of mitosis. Based on these findings, we examined the protein expression levels of cyclins and CDKs in HeLa cells upon treatment with DM. At 24 h after DM treatment, a clear concentration-dependent decrease in protein expression was observed for cyclin D1 and cyclin D3. While other cyclins and CDKs did not show significant concentration-dependent effects, a reduction in their expression was evident at the highest treatment concentration (0.5 mg/ml) (Fig. 4*A*).

After 48 h of DM treatment, all cyclins exhibited a concentration-dependent decrease in protein expression levels, and CDKs also showed a marked reduction in protein expression at the highest treatment concentration (Fig. 4*B*). Next, with the DM treatment concentration fixed at 0.50 mg/ml, a time-course analysis up to 24 h was conducted. It was found that cyclin D1 and cyclin D3 exhibited a more rapid decrease in protein expression levels compared to other cyclins, with decreases of about 98% and 40%, respectively, within 24 h of DM treatment. Notably, cyclin D1 showed a reduction in protein levels as early as 3 h after DM treatment, followed by a dramatic decrease over time (Fig. 4*C*). Regarding CDK (CDK4) and cyclin-dependent kinase inhibitors (CKIs, p15 INK4b, and p57 Kip2), no reduction in their protein expression levels was observed within 24 h. CKIs are known to comprise two main families: the INK4 family, which includes p16INK4a, p16INK4b, p16INK4c, and p16INK4d, and the Cip/Kip family, consisting of p21Waf/Cip1, p27Kip1, and p57Kip2 (14). While the effects on p16INK4c and p16INK4d were not examined, Western blotting analysis revealed no impact on the protein levels of the other molecules from both families within 24 h of DM treatment (Fig. 4*C*). Based on these experiments, it was found that while DM treatment led to a decrease in the protein levels of various cell cycle regulatory factors after 48 h, the effect was more pronounced on cyclins compared to CDKs and CKIs. The rapid decrease in the protein expression levels of cyclin D1 and cyclin D3 induced by DM was an unanticipated and noteworthy finding. Cyclin D2 exhibited markedly lower gene expression levels compared to cyclin D1 and cyclin D3. Furthermore, no significant changes were observed in cyclin D2 protein levels, particularly in response to DM treatment. Therefore, we focused our subsequent investigations on cyclin D1 and cyclin D3.

Next, to examine whether the downregulation of cyclin D1 or cyclin D3 affects the inhibitory effect of DM on cell viability, gene knockdown experiments were performed. Knockdown of cyclin D1 or cyclin D3 by siRNA resulted in a more than 90% reduction in gene expression levels compared with the control (Fig. 4*D*). Under these conditions, cell viability was assessed 48 h after DM treatment (Fig. 4*E*). Knockdown of cyclin D1 alone markedly reduced cell viability, even under DM-free conditions. Furthermore, when the difference between No DM and DM+ was examined, the DM-induced reduction in viability was abolished under cyclin D1 knockdown, and the magnitude of the DM effect (Δdiff) was significantly different from that of the control group. In contrast, cyclin D3 knockdown still showed a significant reduction upon DM treatment; however, the magnitude of the DM effect compared with the control group was not significant. Although a statistically clear attenuation of the DM effect was not confirmed, the changes in mean values suggested a trend toward weaker suppression, indicating that cyclin D3 may partially contribute to the action of DM.

Taken together, these results suggest that the downregulation of cyclin D1 or D3 is not merely a consequence of DM action but also a cause mediating its inhibitory effect on cell viability. Accordingly, subsequent analyses focused on cyclin D1 and cyclin D3 to investigate the mechanisms of their degradation.

### Caspase activation mediates DM-induced apoptosis, whereas downregulation of cyclin D1 and cyclin D3 occurs independent of caspases

**Figure 5:**
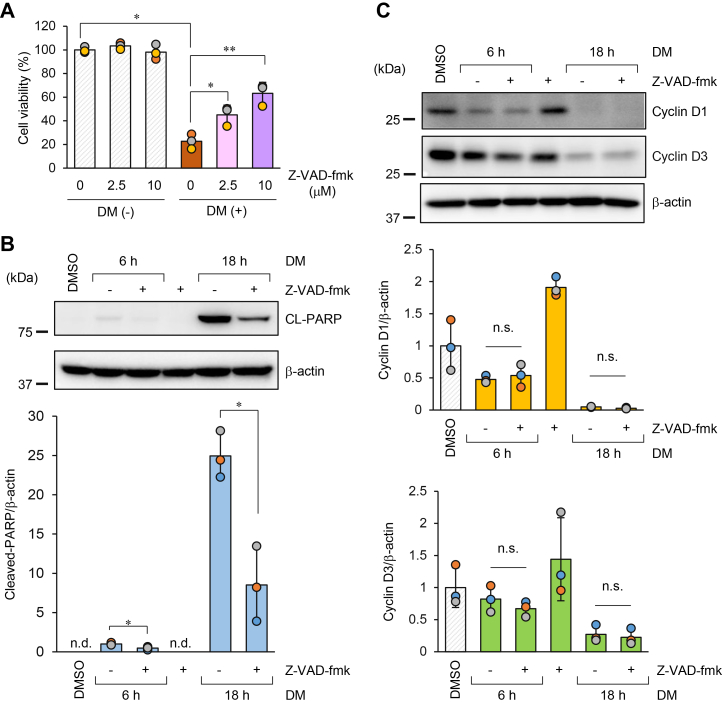
**Evaluation of caspase involvement in DM-induced reduction in cell viability and downregulation of cyclin D1 and D3.***A*, Effect of a pan-caspase inhibitor (Z-VAD-fmk) on DM-induced growth inhibition, as assessed by a cell viability assay. HeLa cells (1 × 10^4^) were seeded in 96-well plates and treated with 0.25 mg/ml DM in the presence of Z-VAD-fmk (2.5 and 10 μM). After 42 h, viable cells were quantified as described in the Experimental procedures section. The graphs show the relative cell viability compared with the control (0 mg/ml DM). Data are presented as mean ± SD (n = 3 per group). Statistical significance was assessed using one-way ANOVA (F(3, 8) = 64.31, *p*= 6.09 × 10^-6^), followed by Dunnett’s multiple comparisons test. The control group (without DM or Z-VAD-fmk) was compared with the DM-treated group. In addition, the DM-treated group was compared with the DM + Z-VAD-fmk groups (2.5 and 10 μM). ∗*p*< 0.05, ∗∗*p*< 0.01. *B*, *C*. Effect of a pan-caspase inhibitor on DM-induced PARP cleavage (*B*) and reduction of cyclin D1 and cyclin D3 protein levels (*C*), as assessed by Western blotting. HeLa cells were treated with Z-VAD-fmk (10 μM) and DM (0.25 mg/ml) simultaneously, and the cells were harvested at 6 h and 18 h. Soluble proteins were extracted and subjected to Western blotting to detect cleaved PARP (*B*), cyclin D1, and cyclin D3 (*C*). β-Actin was used as a loading control. Band intensities were quantified as described in the Experimental procedures section. The relative expression levels of each protein compared with the 6 h DM treatment without Z-VAD-fmk (*B*) or the DMSO control (*C*) are graphed. Data are presented as mean ± SD (n = 3 per group). These experiments were independently repeated at least twice, and representative data are shown. Statistical significance was assessed by Welch’s *t* test, comparing the DM treatment without Z-VAD-fmk with the DM + Z-VAD-fmk groups at 6 h and 18 h, respectively. ∗*p*< 0.05.

To determine whether DM-induced cell death occurs through caspase-dependent apoptosis, we employed the pan-caspase inhibitor Z-VAD-fmk. As shown in Figure. 5*A*, Z-VAD-fmk significantly attenuated the DM-induced reduction in cell viability in a dose-dependent manner. Z-VAD-fmk alone did not exert a significant effect on cell viability. In addition, treatment with DM alone resulted in the cleavage of poly(ADP-ribose) polymerase (PARP; 116 kDa → 89 kDa), a substrate of caspases, together with the appearance of cleaved caspase-3 (17/19 kDa) and caspase-7 (20 kDa), which is indicative of their activation (Fig. S3). Consistently, a time-dependent increase in PARP cleavage was observed (Fig. 5*B*), and this cleavage was significantly suppressed by Z-VAD-fmk. These results strongly support the notion that DM induces caspase-dependent apoptosis.

We next examined whether the DM-induced reduction in cyclin D1/D3 protein levels depends on caspase activation. Western blot analysis revealed that Z-VAD-fmk failed to restore the DM-induced reduction of cyclin D1 and cyclin D3 (Fig. 5*C*). Collectively, these findings suggest that while DM activates caspase-dependent apoptosis, the reduction of cyclin D1 and cyclin D3 is likely mediated by a caspase-independent mechanism.

### Examination of the potential involvement of the Wnt/GSK-3β/β-catenin signaling pathway in the downregulation of cyclin D expression

The elucidation of the mechanisms underlying the downregulation of cyclin D1 and cyclin D3 expression by DM is crucial for understanding the pathway leading from cell cycle disturbance to cell death. Thus, we focused our analysis on the gene expression mechanism and the degradation mechanism to elucidate the regulatory mechanisms governing the reduced protein expression of cyclin D1 and cyclin D3. When analyzing gene expression levels using real-time quantitative PCR (RT-qPCR), melanin in the samples affected the process as a PCR inhibitor. Therefore, we removed DM from the RNA samples using a commercially available kit and performed a gene expression analysis with the purified RNA.

**Figure 6:**
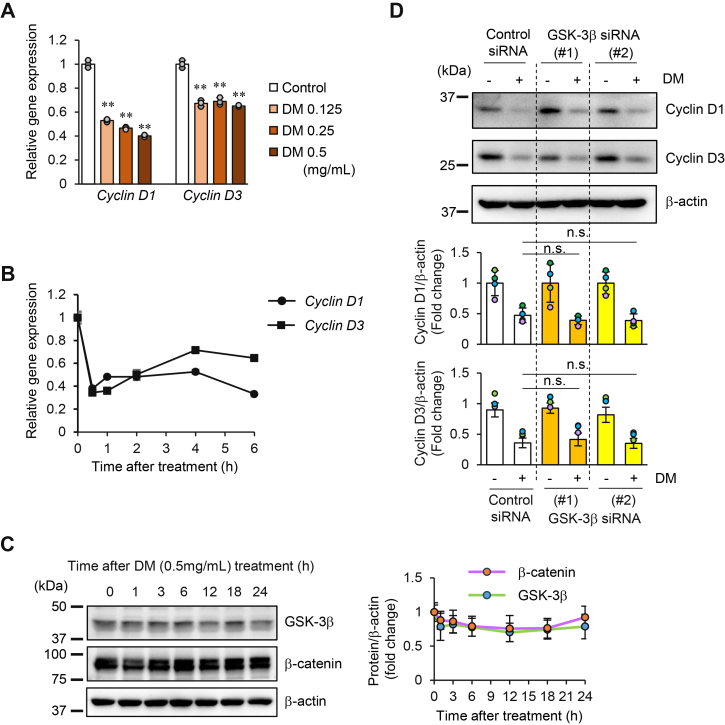
**Effects of DM on cyclin D1 and D3 gene expression and the involvement of the GSK3-β/β-catenin pathway**. *A*, concentration-dependent changes in cyclin D1 and cyclin D3 mRNA levels in HeLa cells treated with DM for 6 h. Relative expression levels analyzed by RT-qPCR and shown compared to the untreated control. Statistical analysis was performed on three biological replicates using one-way ANOVA followed by Dunnett’s test, comparing each treatment group to the control (0 mg/ml DM) for each gene to determine statistical significance. Cyclin D1: F(3, 8) = 657.3, *p*= 6.55 × 10^-10^; Cyclin D3: F(3, 8) = 124.3, *p*= 4.75 × 10^-7^. ∗∗*p*< 0.01. *B*. Time-dependent changes in cyclin D1 and cyclin D3 mRNA levels following DM treatment (0.5 mg/ml). Relative expression levels analyzed by RT-qPCR and shown compared to the 0-h time point. *C*, Western blot analysis of GSK-3β and β-catenin expression in cells treated with DM (0.5 mg/ml) for the indicated times. Representative Western blot images are shown (left panel). β-Actin was used as a loading control. Band intensities of GSK-3β and β-catenin were quantified using ImageJ software, as described in the Experimental procedures section, from three independent biological replicates, normalized to β-actin, and expressed as fold change relative to the 0 h control (vehicle: water). Quantified data are shown as graphs (*right panel*). Data are presented as mean ± SD (n = 3 per group). No significant differences were observed between time points for either protein (one-way ANOVA; GSK-3β: F(6, 14) = 1.006, *p*= 0.459; β-catenin: F(6, 14) = 1.24, *p*= 0.344). *D*, effect of GSK-3β knockdown on DM-induced cyclin D1 and cyclin D3 protein expression. HeLa cells were transfected with two GSK-3β siRNAs (#1, #2) for 24 h, then treated with 0.5 mg/ml DM for 24 h. Protein levels were analyzed by Western blotting with β-actin as normalizer. Band intensities were quantified as described in the Experimental procedures section. The graph shows relative expression levels normalized to untreated samples for each siRNA condition. Statistical analysis was performed on four biological replicates using one-way ANOVA followed by Dunnett’s test, comparing each siRNA group to the control siRNA to determine statistical significance. cyclin D1: F(2, 9) = 0.889, *p*= 0.444; cyclin D3: F(2, 9) = 1.605, *p*= 0.254. n.s., not significant.

After 6 h of DM treatment at various concentrations, a significant concentration-dependent decrease in the expression of these genes was observed (Fig. 6*A*). We then examined the gene expression levels from 30 min to 6 h after DM treatment, and a significant decrease in expression of both cyclin D1 and cyclin D3 was observed at 30 min. However, neither gene exhibited a linear decreasing trend in gene expression (Fig. 6*B*).

Next, we examined the effects of DM on the transcriptional regulatory signaling pathways of cyclin D1 and cyclin D3. Since the gene expression of cyclin D1 and cyclin D3 is positively regulated by the transcription factor β-catenin (16, 17, 18), we investigated the Wnt/GSK-3β/β-catenin signaling pathway. In the Wnt/GSK-3β/β-catenin pathway, the Wnt protein binds to the Frizzled receptor, initiating signal transduction, and β-catenin, accumulates in the nucleus through the inactivation of GSK-3β. Specifically, when GSK-3β is activated, it phosphorylates β-catenin, and the phosphorylated β-catenin is ubiquitinated and degraded. Conversely, when GSK-3β is inactivated, β-catenin escapes phosphorylation, translocates to the nucleus, and forms a complex with the transcription factor LEF1/TCF, promoting the transcription of cyclin D1 (19). Additionally, β-catenin promotes the transcription of cyclin D3 through the activation of c-Jun (17).

Therefore, since the decrease in the expression levels of β-catenin and GSK-3β leads to a reduction in the expression of cyclin D1 and cyclin D3, we examined the protein expression levels of each up to 24 h after DM treatment. The results of Western blotting showed that DM treatment did not cause a decrease in the protein expression levels of either GSK-3β or β-catenin (Fig. 6*C*). These results suggested that the decrease in cyclin D1 and cyclin D3 expression was not due to a decrease in GSK-3β or β-catenin. Furthermore, GSK-3β directly phosphorylates cyclin D1 and cyclin D3, resulting in them becoming targets for proteasomal degradation (19, 20). To confirm whether GSK-3β is involved in the decreased expression of cyclin D induced by DM, we performed gene knockdown experiments. Using two different siRNAs, we confirmed that GSK-3β gene expression was suppressed by more than 95% in both cases, with protein expression reduced by more than 58% (Fig. S4*A*). By fixing the DM concentration at 0.5 mg/ml and repeating the experiments multiple times, no significant difference was found in the decrease from the baseline level compared to the control for either cyclin D1 or cyclin D3 (Fig. 6*D*). These results suggest that the Wnt/GSK-3β/β-catenin signaling pathway is unlikely to be involved in the decreased expression of cyclin D1 and cyclin D3 induced by DM.

**Figure 7:**
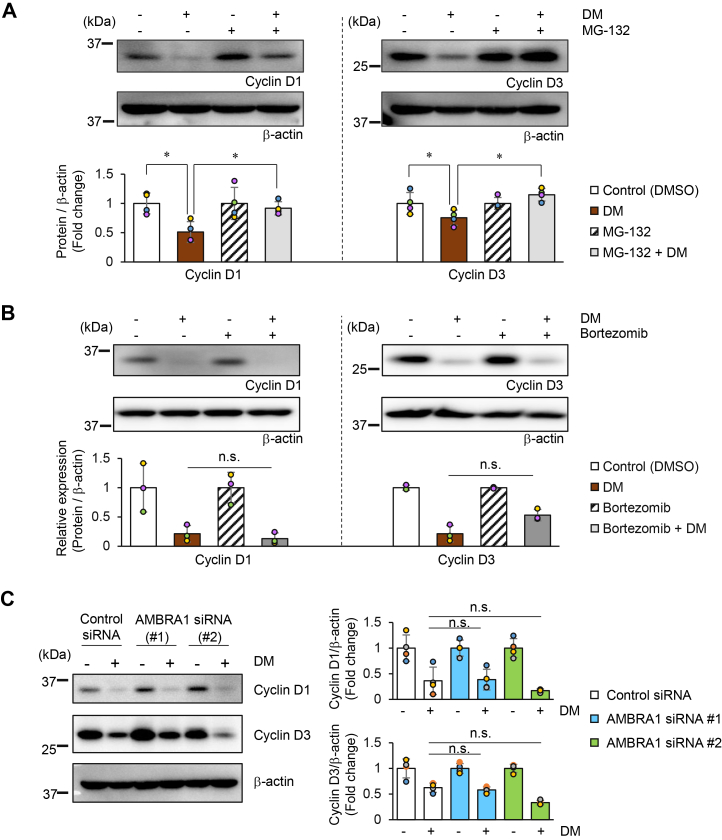
**Involvement of protein degradation systems in DM-induced cyclin D downregulation.** HeLa cells were pretreated with MG-132 (5 μM) (*A*) or bortezomib (10 μM) (*B*) for 1 h, followed by treatment with 0.5 mg/ml DM for 6 h. Protein expression levels of cyclin D1 and cyclin D3 were analyzed by Western blotting. β-actin was used as a loading control. Band intensities were quantified as described in the Experimental procedures section. The graphs show the relative expression levels of each cyclin D normalized to their respective controls in both the inhibitor-treated and untreated groups. *C*. HeLa cells with AMBRA1 knockdown using two different siRNAs were treated with 0.5 mg/ml DM for 6 h. Protein levels of cyclin D1 and cyclin D3 were analyzed by Western blotting. Band intensities were quantified as described in the Experimental procedures section. Graphs show the relative expression levels normalized to untreated samples for each siRNA-transfected cell group. Statistical analysis was performed on four (*A* and *C*) or three (*B*) biological replicates using the one-tailed Mann–Whitney U test to determine statistical significance (∗*p*< 0.05). n.s.: not significant.

While DM treatment decreased the gene expression levels of cyclin D1 and cyclin D3, these did not continue to decrease linearly up to 6 h of treatment, and a certain level of expression was maintained. However, since the protein expression levels (especially cyclin D1) decreased in a time-dependent manner, we next investigated the possibility of enhanced degradation of cyclin D1 and cyclin D3 proteins. The ubiquitin–proteasome pathway is well known to contribute to the degradation mechanism of cyclin D (21). Therefore, we examined this using MG-132, a proteasome inhibitor that effectively inhibits the protein degradation activity of the 26S proteasome complex (22, 23). MG-132 strongly inhibited the degradation of both cyclin D1 and cyclin D3 induced by DM (Fig. 7*A*). To confirm this, we performed a similar examination using bortezomib, another proteasome inhibitor that targets the 20S proteasome (24). Contrary to our expectation, bortezomib did not inhibit the degradation of cyclin D1 and cyclin D3 induced by DM (Fig. 7*B*).

To further investigate whether the ubiquitin–proteasome system is involved in the DM-induced degradation of cyclin D1 and cyclin D3, we focused on AMBRA1, which has recently been reported as a major regulator of cyclin D ubiquitination (25, 26, 27). AMBRA1 functions as a substrate receptor for the E3 ubiquitin ligase complex and promotes ubiquitination and degradation of D-type cyclins. We performed AMBRA1 knockdown experiments to examine whether the enhanced degradation of cyclin D1 and cyclin D3 induced by DM is a result of promoting the ubiquitination of cyclin D. We confirmed that two different siRNAs targeting AMBRA1 suppressed AMBRA1 gene expression by more than 90% in HeLa cells, with protein expression reduced by more than 70% (Fig. S4*B*). As a sufficient decrease in the protein levels of cyclin D1 and cyclin D3 was observed 6 h after DM treatment (Fig. 4*C*), we reexamined the protein expression levels of both proteins at 6 h after DM treatment using cells transfected with two different AMBRA1 siRNAs. When compared to the respective expression levels in the untreated condition, the extent of decrease in the expression levels of cyclin D1 and D3 did not significantly differ between siRNA-transfected and non-transfected cells (Fig. 7*C*). These results suggest that the decrease in the expression of cyclin D1 and D3 induced by DM may not involve the activity of AMBRA1.

### Differential involvement of calpain in DM-induced cyclin D degradation and apoptosis signaling

**Figure 8:**
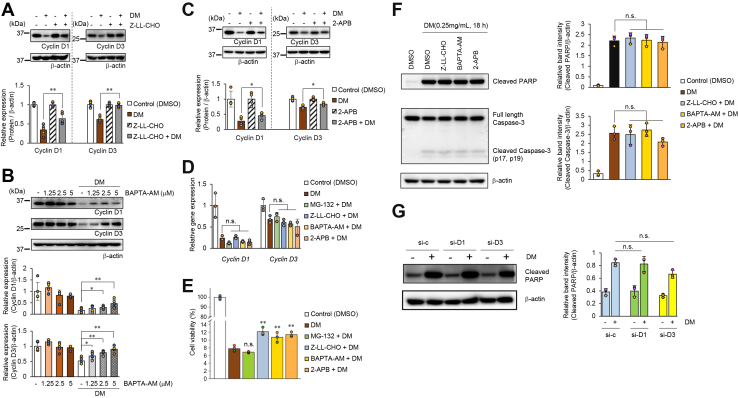
**Differential involvement of calpain in DM-induced cyclin D degradation and apoptosis signaling**. *A*, HeLa cells were pretreated with Z-LL-CHO (10 μM) for 1 h, followed by treatment with 0.5 mg/ml DM for 6 h. Protein expression levels of cyclin D1 and cyclin D3 were analyzed by Western blotting. β-actin was used as a loading control. Band intensities were quantified as described in the Experimental procedures section. Graphs show the relative expression levels of each cyclin D normalized to their respective untreated controls in both the Z-LL-CHO-treated and untreated groups. Statistical analysis was performed on five biological replicates using the one-tailed Mann–Whitney U test to determine statistical significance (∗∗*p*< 0.01). *B*, HeLa cells were pretreated with BAPTA-AM at the concentrations indicated for 1 h, followed by treatment with 0.5 mg/ml DM for 6 h. Protein expression levels of cyclin D1 and cyclin D3 were analyzed by Western blotting. β-actin was used as a loading control. Band intensities were quantified as described in the Experimental procedures section. After normalization to the loading control, graphs show the relative expression levels of each cyclin D compared to the control without BAPTA-AM and DM treatment. Statistical analysis was performed on five biological replicates using the one-tailed Mann-Whitney U test to determine statistical significance (∗*p*< 0.05, ∗∗*p*< 0.01). *C*, HeLa cells were pretreated with 2-APB (50 μM) for 2 h, followed by treatment with 0.5 mg/ml DM for 6 h. Protein expression levels of cyclin D1 and D3 were analyzed by Western blotting. β-actin was used as a loading control. Band intensities were quantified as described in the Experimental procedures section. Graphs show the relative expression levels of each cyclin D normalized to their respective untreated controls in both 2-APB-treated and untreated groups. Statistical analysis was performed on four biological replicates using the one-tailed Mann–Whitney U test to determine statistical significance (∗*p*< 0.05). *D*. HeLa cells were pretreated with MG-132 (5 μM, 1 h), Z-LL-CHO (10 μM, 1 h), BAPTA-AM (5 μM, 1 h), or 2-APB (50 μM, 2 h), followed by treatment with 0.5 mg/ml DM for 6 h. RNA was extracted, and cyclin D1 and D3 gene expression was measured by RT-qPCR. GAPDH gene was used as a normalizer, and the relative gene expression levels compared to the DMSO control samples were graphed. Statistical analysis was performed on four biological replicates using the one-tailed Mann–Whitney *U* test to determine statistical significance. n.s.: not significant. *E*, HeLa cells (1 × 10^4^) were seeded in 96-well plates and pretreated with MG-132 (5 μM, 1 h), Z-LL-CHO (10 μM, 1 h), BAPTA-AM (5 μM, 1 h), or 2-APB (50 μM, 2 h). Cells were then treated with DM (0.5 mg/ml) in the presence of each inhibitor. After 48 h, viable cells were quantified as described in the Experimental procedures section. The graphs show the relative cell viability compared with the DM-treated control (0.5 mg/ml DM). Data are presented as mean ± SD (n = 3 per group). Statistical significance was assessed using one-way ANOVA (F(4, 13) = 37.67, *p*= 4.94 × 10^-7^), followed by Dunnett’s multiple comparisons test. Each inhibitor-treated group was compared with the DM-treated control group. ∗∗*p*< 0.01, n.s., not significant. *F*, effects of calpain pathway inhibitors on DM-induced apoptotic signaling. HeLa cells were pretreated with Z-LL-CHO (10 μM, 1 h), BAPTA-AM (5 μM, 1 h), or 2-APB (50 μM, 2 h), followed by treatment with DM (0.25 mg/ml) for 18 h. The inhibitors were dissolved in DMSO, and an equivalent volume of DMSO was used as vehicle control. Cleaved PARP, full-length caspase-3, and cleaved caspase-3 were analyzed by Western blotting; β-actin was used as a loading control. Band intensities were quantified using ImageJ software, as described in the Experimental procedures section, normalized to β-actin, and expressed as relative values. Statistical analysis was performed using three independent biological replicates with a two-tailed Mann–Whitney U test. Statistical significance was determined relative to DM-treated cells in the absence of inhibitors. n.s., not significant. *G*. Effect of cyclin D1 or cyclin D3 knockdown on DM-induced PARP cleavage. HeLa cells were transfected with control siRNA, cyclin D1 siRNA, or cyclin D3 siRNA using a reverse transfection method. Twenty-four hours after transfection, the cells were treated with DM (0.25 mg/ml) or vehicle (water) for 18 h. Cleaved PARP was analyzed by Western blotting. β-actin was used as a loading control. Band intensities were quantified using ImageJ software, as described in the Experimental procedures section, normalized to β-actin, and expressed as relative values. Statistical analysis was performed using three independent biological replicates with a two-tailed Mann–Whitney U test. Statistical significance was determined relative to control siRNA-treated cells with DM treatment. n.s., not significant.

Why did MG-132 suppress the decrease in the expression of cyclin D1 and cyclin D3 due to DM? To address this question, we focused on another function of MG-132: its role as a calpain inhibitor, which is absent in bortezomib. Calpains belong to the non-lysosomal cysteine protease family and control downstream signaling by carrying out the limited degradation of substrates in a Ca^2+^-dependent manner. Cyclin D1 and cyclin D3 are reportedly subjected to post-translational regulation by calpains (28, 29). Therefore, we investigated how the degradation of cyclin D1 and D3 by DM may be mediated by calpains, using a potent calpain inhibitor, benzyloxycarbonyl-L-leucyl-L-leucinal (Z-LL-CHO), which does not affect proteasome activity (30). Pretreatment with Z-LL-CHO significantly suppressed the degradation of cyclin D1 and D3 induced by DM (Fig. 8*A*). In particular, the degradation of cyclin D3 by DM was almost completely inhibited.

Since calpain activity is calcium-dependent, we next examined whether a similar effect could be obtained using BAPTA-AM (an intracellular calcium chelator) to further confirm the contribution of calpains. BAPTA-AM significantly inhibited the DM-induced degradation of cyclin D1 and cyclin D3 in a concentration-dependent manner (Fig. 8*B*). This result suggests an enhancement of intracellular calcium production. Therefore, we investigated the involvement of the endoplasmic reticulum (an intracellular organelle and a crucial calcium store) as a source of intracellular calcium release induced by DM.

It is known that the major pathways for calcium release from the endoplasmic reticulum are mediated by the inositol 1,4,5-trisphosphate receptor (IP_3_R) and ryanodine receptor (RyR). However, HeLa cells are non-excitable cells in which RyR expression is considered to be limited, and no functional response has been reported, whereas IP_3_R is widely expressed and serves as the principal pathway for calcium signaling (30, 31).

Therefore, in this study, we focused on the IP_3_R-mediated pathway. 2-aminoethyl diphenylborinate (2-APB) is a well-known inhibitor of IP_3_R, and treatment with 50 μM 2-APB almost completely abolished histamine-induced calcium release (Fig. S5). We thus investigated whether the DM-induced degradation of cyclin D1 and cyclin D3 was affected by treatment with 50 μM 2-APB. As shown in Figure. 8*C*, 2-APB significantly, albeit modestly, inhibited the DM-induced degradation of cyclin D1 and cyclin D3.

We previously demonstrated that DM suppressed the gene expression of cyclin D1 and cyclin D3 (Fig. 6, *A* and *B*). We then investigated whether the various inhibitors that effectively suppressed the DM-induced reduction of cyclin D1 and cyclin D3 at the protein level could also inhibit the decrease in gene expression. To address this question, we employed quantitative PCR to examine the effects of the previously utilized inhibitors on cyclin D1 and cyclin D3 gene expression.

Our results revealed that none of the inhibitors, at concentrations effective in suppressing the DM-induced reduction of cyclin D1 and cyclin D3 at the protein level, restored the DM-induced downregulation of these genes. In fact, some inhibitors further enhanced the DM-induced reduction in gene expression (Fig. 8*D*). These findings suggest that the primary effect of the inhibitors is on the suppression of protein degradation, rather than on gene expression.

Does the inhibition of cyclin D degradation attenuate the inhibitory effect of DM on cell viability? To address this question, we examined DM-induced growth inhibition in the presence of inhibitors that were effective in suppressing cyclin D1 and cyclin D3 degradation. The calpain inhibitor Z-LL-CHO, the calcium chelator BAPTA-AM, and the IP_3_R inhibitor 2-APB significantly attenuated the inhibitory effect of DM on cell viability. In contrast, MG-132 did not show such an effect (Fig. 8*E*). Because MG-132 is a potent inhibitor of both the proteasome and calpain and is also known to induce reactive oxygen species (ROS)-dependent apoptosis on its own (31), it is likely that cytotoxic effects other than cyclin D degradation inhibition were manifested. Taken together, these results suggest that the inhibitory effect of DM on cell viability depends on the degradation of cyclin D1 and cyclin D3.

We next investigated whether these calpain pathway inhibitors suppress DM-induced apoptotic signaling. Even when cells were treated with each inhibitor at concentrations sufficient to block cyclin D1 and cyclin D3 degradation, no significant inhibition of caspase-3 cleavage or PARP cleavage was observed (Fig. 8*F*). We further examined the effect of the individual knockdown of cyclin D1 or D3 on DM-induced PARP cleavage. PARP cleavage induced by DM was not suppressed under either knockdown condition (Fig. 8*G*).

Based on these results, we conclude that the DM-induced reduction of cyclin D1 and cyclin D3 at the protein level is largely attributable to calcium-dependent protein degradation involving calpain. Furthermore, our findings suggest that calcium derived from IP_3_ receptors in the endoplasmic reticulum may partially contribute to this process. Additionally, the calpain-mediated degradation of cyclin D1 and cyclin D3 induced by DM appears to occur independently of apoptotic signaling, suggesting that these two processes represent distinct downstream pathways of DM action.

### Antitumor effects of DM in an allograft model

Finally, we investigated whether the oral administration of DM exerts antitumor effects using an animal model. To ensure accurate dosing, we employed an oral gavage method for direct administration into the stomach. Following the OECD Guideline TG407 *Repeated Dose 28-days Oral Toxicity Study in Rodents*, we conducted a repeated dose oral toxicity study: 1000 mg/kg/2 days administered every other day. As no effects on body weight or food consumption were observed, and no mortality occurred, even after continuing the administration for at least 28 days (Fig. S6, *A* and *B*), we did not proceed with a limit test at higher concentrations. For the allograft model, we employed the implantation of B16F10 mouse melanoma cells into nude mice.

**Figure 9:**
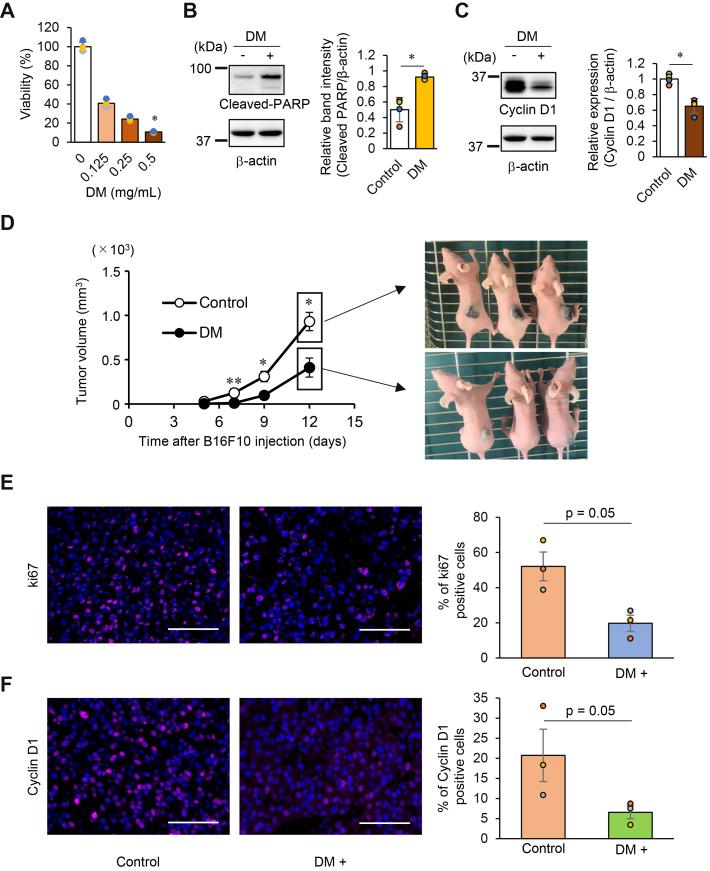
**Effects of DM on B16F10 melanoma cells *in vitro* and *in vivo*.***A*, B16F10 cells were seeded at 1 × 104 cells per well and immediately treated with the indicated concentrations of DM. After 48 h, cell viability was quantified, as described in the Experimental procedures section, and expressed relative to the control (0 mg/ml DM, water). The data represent three to four biological replicates. Statistical analysis was performed using the Kruskal–Wallis test, followed by Dunn’s test with Bonferroni correction. ∗*p*< 0.05 *versus* control (0 mg/ml DM). *B* and C, B16F10 cells were treated with DM (0.5 mg/ml) or water for 24 h, and the expression of cleaved PARP (*B*) and cyclin D1 (*C*) was assessed by Western blotting. Band intensities were quantified using ImageJ software and normalized to β-actin. *B*. Data are expressed as relative band intensity (cleaved PARP/β-actin; n = 4). *C*, data are expressed as fold change relative to the control (cyclin D1/β-actin; n = 4). Statistical significance was determined by the Mann–Whitney U test. ∗*p*< 0.05. *D*, B16F10 cells (2 × 10^5^cells/0.1 ml) were subcutaneously injected into the dorsal region of 9-week-old nude mice. DM (50 mg/ml, 0.2 ml) was orally administered every 2 to 3 days. Once the tumors became visible, their long and short diameters were measured. Tumor volumes were estimated using the method described in the Experimental procedures section and graphed. Representative images of tumors in mice 12 days post-B16F10 cell injection are shown. Statistical analysis was performed on five biological replicates using the Mann–Whitney U test to determine statistical significance (∗*p*< 0.05, ∗∗*p*< 0.01). *E*, *F*, detection of proliferation markers by immunofluorescence staining. Tumor masses were excised on day 12 after B16F10 B16F1 cell implantation, and expression of Ki-67 (*E*) and cyclin D1 (*F*) was detected by immunofluorescence staining as described in the Experimental procedures section. Representative fluorescence images are shown. Image analysis and quantification were performed using ImageJ software. For each group (n = 3), mean values from randomly selected fields per animal were plotted as bar graphs (total images analyzed: 6–8 per group). Bar graphs show the percentage of Ki-67-positive cells (*E*) or cyclin D1-positive cells (*F*) normalized to DAPI-positive cells (% of positive cells = marker^+^/DAPI^+^× 100). Statistical significance was determined by the one-tailed Mann–Whitney U test. *p*= 0.05. Scale bar = 100 μm.

Although B16F10 cells are melanin-producing cells, we examined whether they were susceptible to DM-induced cell death. As observed in the HeLa cells, treatment with DM significantly suppressed the viability of B16F10 cells in a concentration-dependent manner (Fig. 9*A*). Regarding apoptotic signaling, DM treatment induced upregulation of cleaved PARP expression (Fig. 9*B*), strongly suggesting activation of the caspase pathway. Consistent with the results obtained in the HeLa cells, a significant downregulation of cyclin D1 expression was also observed (Fig. 9*C*).

Using a nude mouse model of B16F10 cell transplantation, we investigated whether oral administration of DM suppresses tumor growth. After transplanting B16F10 cells into the dorsal region of nude mice, DM was administered directly into the stomach at a dose of 500 mg/kg once every 2 days, and tumor volumes were measured.

Interestingly, oral administration of DM significantly suppressed the volume increase in the transplanted cancer (Fig. 9*D*). Tumor masses were excised on day 12 after transplantation, and the expression of Ki-67 and cyclin D1, both markers of cell proliferation, was examined by immunofluorescence staining. In the DM-treated group, the percentages of Ki-67-positive cells and cyclin D1-positive cells were both reduced compared to the control, and the differences were statistically significant (Mann–Whitney U test, *p*= 0.05 for both; Fig. 9, *E* and *F*). These results suggest that DM might be absorbed from the intestine and enter the systemic circulation. The mechanism by which orally administered DM suppresses tumor growth remains to be elucidated, and future studies should investigate its intestinal absorption and systemic pharmacokinetics using more rigorous *in vivo* methods.

## Discussion

This study presents a novel approach to cancer treatment through the development and characterization of a highly water-soluble synthetic melanin (DM). Our research aimed to elucidate the anticancer properties of DM and its underlying molecular mechanisms, with the ultimate goal of establishing its potential as a new therapeutic agent in oncology. The synthetic process for DM, starting from L-dopa, yielded a melanin with distinct structural and chemical properties compared to commercial melanins. DM exhibited a smooth, flat morphology rather than aggregated granules and contained sodium without chloride, in contrast to commercial products. These features likely underlie its enhanced water solubility and potential biological utility. Our preparation method produced a polymer with heterogeneous chain lengths, as reflected by a broad molecular weight distribution.

DM demonstrated inhibitory effects on the viability of all tested cancer cell lines. Fractionation analysis revealed that the activity was predominantly associated with high-molecular-weight polymers exceeding approximately 30 kDa, indicating that a minimum molecular weight threshold is critical for the antitumor effect. This size-dependent activity suggests that the therapeutic mechanism may involve multivalent interactions with cellular targets. Although the isolation of a homogeneous active compound would be ideal for drug development, we used unfractionated DM in this mechanistic study because the active fraction itself comprises a heterogeneous mixture of polymeric species. Beyond growth inhibition, DM effectively suppressed the migration and three-dimensional growth of HeLa cells, demonstrating broad anticancer activity.

To assess whether the observed effects were specific to our DM preparation, we examined commercially available melanin processed by ultrasonic disruption. This material also inhibited cancer cell viability compared to DM (Fig. S1). However, even after ultrasonic treatment, commercially available melanin products remain highly heterogeneous mixtures with broad particle-size distributions. It is possible that after application to cells, some larger particles adhered to the cell surface and were subsequently removed during the washing steps. This may have resulted in an apparently stronger inhibitory effect on cell viability compared with DM in the quantitative analysis. Moreover, such heterogeneity presents significant challenges for pharmaceutical development, particularly regarding reproducibility and bioavailability.

In contrast, DM can be produced using a simple and cost-effective process and is characterized by high water solubility—a critical advantage for systemic delivery. These characteristics position DM as a more promising candidate than conventional melanin products for therapeutic development. Future efforts should focus on identifying the specific structural features within the >30 kDa fraction that confer anticancer activity and on developing scalable purification strategies.

Analysis of the HeLa-Fucci cells revealed that DM treatment for 48 h reduced the proportion of G_1_ and G_1_/S cells while increasing S/G_2_/M cells, indicating S/G_2_/M arrest. The cells subsequently lost Fucci fluorescence and motility before undergoing cell death. This appears inconsistent with the later decrease in cyclin D, which is generally linked to G_1_ arrest. However, in cells lacking functional pRB, such as HeLa cells, G_1_ arrest is inefficient under reduced proliferative signaling or DNA damage, and G_2_ arrest occurs instead (32). Furthermore, it has been reported that, depending on the stimuli, downregulation of cyclin D1 can induce G_2_/M rather than G_1_ arrest (33, 34), further supporting this possibility. Taken together, these findings suggest that DM-induced growth suppression in HeLa cells involves atypical cell cycle checkpoint mechanisms, leading to G_2_/M arrest. Detailed characterization of these arrest patterns will be important for future mechanistic studies.

Although apoptosis-related events, such as caspase activation and PARP cleavage, were detected, cyclin D1 and D3 degradation occurred independently of caspase activity. Notably, typical apoptotic body formation was rarely observed, and cells lost viability abruptly after one or two divisions. This atypical cell death pattern, characterized by caspase-independent cyclin degradation and the absence of classic apoptotic morphology, appears distinct from conventional apoptosis. Further characterization of this cell death mechanism will require systematic comparison with established pathways, including autophagy, ferroptosis, necrosis, and pyroptosis, which we are currently investigating.

An ideal anticancer agent selectively targets cancer cells while sparing normal cells. Preliminary observations suggested that HaCaT and WI-38 cells retained motility under DM treatment; however, cell viability and proliferation were not formally quantified in these cell lines, and migration was not examined. Therefore, the selective toxicity of DM toward cancer cells over normal cells remains to be rigorously established. Indeed, further studies using a broader panel of normal cell types with quantitative assessments of viability, proliferation, and migration are needed to fully evaluate the potential of DM as a selective anticancer agent.

To explore the molecular basis of DM’s effects, we examined cell cycle regulators and found that DM rapidly reduced cyclin D1 and D3 levels, aligning with the critical role of D-type cyclins in cancer progression (21) and the therapeutic relevance of CDK4/6 inhibitors (35). Functional studies showed that cyclin D1 reduction was primarily responsible for growth inhibition, while cyclin D3 contributed secondarily. Thus, DM’s inhibitory effect on cell viability appears to be mediated mainly through suppression of cyclin D1, with cyclin D3 playing a supportive role.

As described above, although DM activated the caspase pathway, the reduction in cyclin D1 and cyclin D3 protein levels was not rescued by treatment with a pan-caspase inhibitor. While caspase-dependent degradation of cyclin E has been reported to play a functional role in apoptosis (36), there have been no reports of caspases directly mediating the degradation of cyclin D. Our results therefore suggest that caspase activation does not directly contribute to the degradation of cyclin D1 and cyclin D3.

Our investigation into the mechanisms underlying DM-induced cyclin D downregulation encompassed both transcriptional and post-translational processes. qRT-PCR analysis demonstrated that DM treatment led to decreased mRNA expression levels of cyclin D1 and cyclin D3. However, the non-linear time-dependent pattern of this suppression suggests the involvement of additional regulatory mechanisms, particularly at the protein level. We initially hypothesized that DM might affect cyclin D expression through modulation of the GSK-3β/β-catenin pathway, a known positive regulator of cyclin D transcription. However, our experiments showed no changes in GSK-3β or β-catenin protein levels upon DM treatment, and GSK-3β knockdown did not attenuate DM’s effects on cyclin D expression, indicating that DM’s mechanism of action is likely independent of the GSK-3β/β-catenin axis. However, cyclin D expression is regulated at multiple levels. At the transcriptional level, in addition to the Wnt/GSK-3β/β-catenin pathway, several signaling pathways are known to regulate cyclin D, including the MAPK/ERK/AP-1, PI3K/Akt, STAT3, and NF-κB pathways. At the post-transcriptional level, suppression of cyclin D expression has been reported through microRNAs that target cyclin D1 and D3 mRNA, including the miR-16 family, miR-34a, and miR-195 (37, 38, 39). Whether the DM-induced reduction in cyclin D mRNA expression occurs through transcriptional or post-transcriptional regulatory mechanisms remains to be elucidated.

Given the rapid decrease in cyclin D protein levels, we investigated the potential involvement of protein degradation pathways. Initial experiments with the proteasome inhibitor MG-132 showed significant attenuation of DM-induced cyclin D1 and cyclin D3 degradation. However, this effect was not replicated with another proteasome inhibitor, bortezomib, suggesting that proteasomal degradation might not be the primary mechanism. Since AMBRA1, a component of the CRL4 E3 ubiquitin ligase complex, is a key regulator of cyclin D degradation (25, 26, 27), we tested its involvement. AMBRA1 knockdown had no effect on DM-induced cyclin D1 or cyclin D3 reduction, further indicating that enhanced ubiquitin ligase activity is not the primary mechanism.

The differential effects of MG-132 and bortezomib led us to consider the involvement of calpains, calcium-dependent proteases also inhibited by MG-132 but not by bortezomib. Indeed, treatment with the calpain inhibitor Z-LL-CHO significantly suppressed the DM-induced downregulation of cyclin D1 and cyclin D3. This finding was further supported by experiments using the calcium chelator BAPTA-AM, which produced similar results. To identify the source of calcium necessary for calpain activation, we investigated the role of endoplasmic reticulum (ER) calcium channels. The IP_3_ receptor inhibitor 2-APB partially but significantly attenuated DM-induced cyclin D1 and cyclin D3 downregulation. These results suggest that calcium release from the ER, likely through IP_3_ receptors, plays a role in DM’s mechanism of action. However, since 2-APB can act on multiple calcium-regulating proteins, including store-operated calcium entry (SOCE), transient receptor potential (TRP) channels, and sarco/endoplasmic reticulum Ca^2+^-ATPase (SERCA) pumps (40), further investigation is required to determine whether IP_3_R alone is involved. In addition, because three IP_3_R isoforms exist (41), it is important to identify which specific isoform(s) contributes to this response.

These inhibitors did not affect DM-induced reductions in cyclin D1 and D3 mRNA levels, confirming that their effects occurred mainly at the protein level through proteolysis. With the exception of MG-132, they significantly attenuated the inhibitory effect of DM on cell viability, which correlated with the recovery of cyclin D1 and D3 protein levels. This supports the hypothesis that the reduction of these cyclins is closely linked to DM's growth-inhibitory activity. MG-132 suppressed calpain-mediated degradation of cyclin D1 and D3, but failed to prevent DM-induced cell death, likely because MG-132 itself induces apoptosis *via* multiple mechanisms, including proteasome inhibition, ER stress activation, ROS production, and induction of p21 and caspase-3 (42, 43).

In addition to its effects on cyclin D degradation, our data indicate that DM-induced apoptosis signaling occurs independent of calpain activity. Inhibition of calpain-mediated cyclin D degradation did not affect caspase activation or PARP cleavage, and knockdown of cyclin D1 or cyclin D3 had no impact on DM-induced apoptosis. These findings suggest that DM engages at least two distinct pathways: a calpain-dependent pathway leading to cyclin D degradation and a calpain-independent pathway leading to apoptosis signaling.

Importantly, these observations indicate that cyclin D degradation alone is not sufficient to account for the apoptotic effects of DM, supporting the notion that multiple mechanisms contribute to its antitumor activity. The ability of DM to activate multiple pathways may contribute to its cytotoxic effects. From a therapeutic perspective, such multitargeted activity could potentially limit the development of drug resistance in cancer cells. However, the relative contributions of these pathways to cell cycle regulation and apoptosis remain to be determined, and further studies are required to clarify their interactions.

A possible mechanism for DM-induced IP_3_R-mediated calcium release may involve pathways similar to those described in pigment transfer. In melanocyte–keratinocyte co-cultures, pigment transfer elevates intracellular Ca^2+^ from internal stores, a process essential for transfer (44). PAR-2, a G protein–coupled receptor, can trigger PLC–IP_3_–mediated Ca^2+^ signaling and has been implicated in the uptake of non-membrane–bound melanin (melanocores) *via* Rac1/Cdc42-dependent phagocytosis, while membrane-bound melanosomes are internalized through a PAR-2–independent route (45, 46).

Importantly, PAR-2 is expressed not only in keratinocytes but also in many epithelial and non-epithelial cells, including HeLa, where it mediates surface recognition and uptake (47, 48). These findings suggest that exogenous DM may activate PAR-2 at the cell surface, triggering PLC–IP_3_ signaling, ER calcium release, and subsequent calpain activation. However, because synthetic DM may differ from native melanin in its properties and recognition, this remains a hypothesis. To test this model, PAR-2 expression in the experimental cell systems should be confirmed, DM-induced PAR-2 activation should be directly demonstrated, and functional analyses with PAR-2 inhibitors should be performed.

To assess DM’s anticancer potential, we performed a preliminary *in vivo* study using a mouse syngeneic tumor model with B16F10 melanoma cells, which produce melanin stored in melanosomes and generally show low toxicity or even cytoprotective effects (49). In contrast, DM is synthesized cell-free and resembles unencapsulated melanocores. Importantly, DM treatment activated caspase-3 in B16F10 cells, indicating that, as in HeLa and other non-melanin–producing cells, DM reduces cell viability and induces cell death through a shared mechanism. These findings suggest that the antitumor activity of melanin depends on its free, unencapsulated form rather than on melanosome-sequestered melanin.

Oral administration of DM suppressed tumor growth, demonstrating antitumor activity *in vivo*. Although the underlying mechanism remains unclear, it is possible that orally administered DM is absorbed through the intestines, enters systemic circulation, and accumulates in tumor tissues, potentially *via* the enhanced permeability and retention (EPR) effect (50). However, further studies are required to clarify the precise mechanisms underlying the *in vivo* efficacy of DM.

While our findings highlight DM’s anticancer potential, several areas require further investigation. Elucidating the precise molecular structure of DM is essential for its pharmaceutical development, and techniques such as mass spectrometry, atomic force microscopy, and cryo-electron microscopy may provide crucial insights into its structure and properties. Our study focused on calpain-mediated proteolysis of cyclin D1 and cyclin D3, but cyclin Ds are also regulated by multiple transcription factors (AP-1, NF-κB, Oct-1, STAT3, E2F, Sp1, ETS, and FOXO) and epigenetic mechanisms, such as mRNA methylation and stability (51, 52, 53, 54, 55). Further investigation of these pathways will help clarify DM’s comprehensive mechanisms. Additionally, humans possess 15 calpain isoforms (56), and the specific isoform(s) responsible for DM-induced cyclin D degradation remain unidentified.

Optimizing delivery methods, such as intratumoral or intravenous administration, may improve efficacy. Comprehensive safety and toxicity studies are essential to establishing DM’s potential as a therapeutic agent. While our current findings suggest differential responses between cancer and non-malignant cells under the conditions tested, further evaluation across a wider range of normal cell types and *in vivo* models is necessary to fully characterize its safety profile.

In conclusion, we identified DM as a novel anticancer agent with significant anticancer potential. Its mechanism—calcium-dependent calpain activation leading to cyclin D degradation—distinguishes it from existing therapies and suggests the involvement of both caspase-dependent and caspase-independent pathways. Preliminary *in vivo* studies have demonstrated antitumor activity, supporting its potential as a therapeutic candidate.

If our hypothesis is correct, similar effects may also be observed with natural melanin in the form of melanocores or other non–protein- or membrane-bound states, an issue that warrants further investigation. Although additional studies are required to further characterize its mechanisms and evaluate its safety and efficacy, DM represents a promising direction in cancer research, with the potential to contribute to future therapeutic strategies.

## Experimental procedures

### Materials and reagents

Unless indicated, all chemicals and drugs were from FUJIFILM Wako Pure Chemical Corporation. Synthetic melanin was purchased from Sigma-Aldrich and MP-Biomedicals.

### Chemical synthesis of a water-soluble melanin (dopa-melanin; DM)

Water-soluble dopa-melanin (DM) was synthesized in-house, as described previously (9). Briefly, 3-(3,4-dihydroxyphenyl)-L-alanine (L-dopa) was dissolved in 0.025 N NaOH and incubated for 3 days at room temperature with constant aeration. The crude DM was precipitated by adding concentrated HCl. The moist precipitate was collected by centrifugation (1000*g*, 5 min), dissolved in deionized water, and precipitated again with concentrated HCl. The DM was purified this way four times and then dissolved in 0.025 N NaOH. The solution was adjusted to pH 7.4, then desalted with a Float-A-Lizer G2 dialysis device (MWCO 20 kDa; Spectrum Laboratories) and lyophilized.

### Analysis of molecular weight distribution

The analysis was conducted by outsourcing to Japan Food Research Laboratories. The DM powder was dissolved in 0.1 M sodium nitrate solution to prepare a 5 mg/ml solution. The solution was filtered through a 0.45 μm membrane filter and analyzed using a high-performance liquid chromatography system (Shodex GPC-101, RI-71S; Resonac Corporation, Tokyo, Japan) with a size exclusion column (TSKgel GMPW_XL_, ϕ7.8 mm × 300 mm; Tosoh Corporation, Tokyo, Japan). The injection volume was 100 μl, and the mobile phase was 0.1 M sodium nitrate at a flow rate of 1.0 ml/min.

### Scanning electron microscopy

Commercial and synthesized melanin powders were observed under a scanning electron microscope (SEM; JSM-IT700HR, JEOL Ltd) equipped with an energy dispersive X-ray spectroscope (EDX) at an acceleration voltage of 15 kV. The powders had been coated with Pt/Pd thin film for morphological observation, whereas their chemical composition was determined without the coating by the EDX measurements at five different areas. Their means and standard deviations were calculated and used for analysis.

### Fourier transform infrared spectrometer

The Fourier transform infrared (FTIR) transmission spectra were obtained with an FTIR spectrometer (FT/IR-6100; JASCO Corp., Tokyo, Japan) by the KBr pellet method. The spectra were acquired averaging 128 scans in the wavenumber range between 4000 cm^−1^ and 400 cm^−1^ with a nominal spectral resolution of 4 cm^−1^.

### Cell line and cell culture

Human cancer cell lines, HeLa (uterine cervix), HepG2 (liver), Jurkat (T lymphocyte), SK-BR-3 (breast) cell lines were purchased from ATCC. Murine melanoma cell lines of B16F10 was obtained from the Cell Resource Center for Biomedical Research at Tohoku University. MCF-7 (breast), HeLa/Fucci, and WI-38 (normal human fibroblast) were provided by the RIKEN BRC (Tsukuba) through the National BioResource Project of MEXT, Japan. HaCaT (39) (normal human keratinocyte) was obtained from CLS Cell Lines Service GmbH (Eppelheim). All cell lines were obtained from authenticated repositories and used without further in-house authentication. *Mycoplasma* testing was not performed; however, cell morphology and growth characteristics were routinely monitored and no abnormalities were observed throughout the experiments. The cells were maintained in Eagle’s minimum essential medium (EMEM) (HeLa, MCF-7, WI-38), DMEM (HaCaT, HeLa/Fucci, HepG2), or RPMI1640 (Jurkat, B16F10) containing 10% heat-inactivated fetal bovine serum (FBS), 100 units/ml penicillin, and 100 μg/ml streptomycin at 37 °C and 5% CO_2_ in a humidified atmosphere. Cells from passages 3 to 15 from their arrival were used for the experiments.

### Immunoblotting

Western blotting was performed according to the previously described method (9) with minor modifications. Briefly, lysates of HeLa cells were separated by SDS-PAGE (10–15 μg protein/lane) on a 5 to 20% polyacrylamide gradient gel (SuperSep Ace) and proteins were transferred to a polyvinylidene difluoride membrane (Merck Millipore). The membrane was blocked with Bullet Blocking One for Western Blotting (Nacalai Tesque Inc) for 10 min at room temperature and then reacted with primary antibody (all antibodies used at 1:1000 dilution) for 18 to 20 h at 4 °C, followed by reaction with the corresponding secondary horseradish peroxidase-conjugated antibody (all antibodies used at 1:1000 dilution) for 1 h at room temperature. The signals were detected using Western Lightning Plus-ECL (PerkinElmer, Waltham, MA). Chemiluminescence was captured using a cooled CCD LightCapture camera system and analyzed using CS Analyzer (version 2.0) software (ATTO, Tokyo, Japan) or ImageJ software (version 1.52a, National Institute of Health). All antibodies used were those for which the manufacturer claimed specificity. The primary antibodies used, their corresponding secondary antibodies, and their respective product numbers are shown in Table S2.

### Cell viability assay

For the cell viability assay, cells (2 × 10^4^) were transferred into 96-well plates and cultured for 1 h. Diluted DM samples were then added and cultured for 24 h. For the cytotoxicity assay, cells (2 × 10^5^) were transferred into 96-well plates and cultured for 24 h. After confirming that the cells were confluent, DM samples were added and cultured for another 24 h. At the end point, cells were washed three times with PBS, and the viable cells were examined with a WST-8 assay using a Cell Counting Kit-8 (Dojindo, Kumamoto, Japan) according to the manufacturer’s protocol.

### Molecular weight fractionation of DM using ultrafiltration membranes

Molecular weight fractionation was performed using ultrafiltration membranes with molecular weight cut-off (MWCO) values of 30 kDa, 10 kDa, and 3 kDa (Merck Millipore, MA, USA) according to the manufacturer’s instructions. Briefly, 4 ml of unfractionated DM solution (50 mg/ml) was added to the filter device and centrifuged at 6000*g* for 30 min using a high-speed centrifuge (Kubota, Japan). The retentate and filtrate were collected into pre-weighed tubes, dried using a SpeedVac (Thermo Fisher Scientific, Waltham, MA), and redissolved in ultrapure water to a final concentration of 50 mg/ml.

HeLa cells were seeded onto culture dishes and grown until confluence. The monolayers were scratched with a tip at the center of the dishes and washed twice with medium. Initial pictures were taken immediately after the scratches. After incubation for 24 h, additional pictures were taken. The gap distance was quantitatively evaluated using ImageJ software (version 1.52a; National Institutes of Health). Results were graphed as relative moving distance over time.

### Migration assay

After FBS starvation, 5 × 10^4^ HeLa cells were seeded in the 24-well upper chamber of a cell culture insert having an 8-μm pore size membrane (BD Lifesciences) in 300 μl of EMEM (1% FBS) with or without DM samples to be tested. After attachment, 800 μl of EMEM (10% FBS) was added to the lower chamber, and the cells were incubated for 24 h. The polyethylene terephthalate membranes separating the upper and lower chambers were fixed with 4% paraformaldehyde in PBS for 20 min and stained with crystal violet solution (0.1%/H_2_O) for 1 h. The stained cells were washed with H_2_O for an additional 1 h. In four randomly selected fields, the cells that migrated to the reverse surface of the membrane were counted by light microscopy at 200 × magnification.

### 3D culture

HeLa cells (1 × 10^3^, 100 μl) were seeded in the PrimeSurface 3D Culture Spheroid plates (Sumitomo Bakelite Co., Ltd) and cultured for 24 h. Then, 100 μl of DM sample twice the final concentration was added. Half the volume of the cultured medium with or without the same concentrations of DM was changed every 3 to 4 days. The cells that formed spheroids were photographed 1 day, 2 weeks, and 3 weeks after seeding. The diameters of each spheroid were analyzed using ImageJ software.

### Fluorescence microscopy and cell cycle analysis

HeLa/Fucci cells (1 × 10^4^, 1 ml) were seeded in the glass-bottom chamber slide (AGC Techno Glass, Shizuoka, Japan) and cultured for 24 h. Then, cells were treated with different concentrations of DM for 24 and 46 h. The medium containing DM was removed, and fresh DMEM medium was added. The fluorescence images were obtained using fluorescence microscopy (BZ-X810; Keyence Corporation, Osaka, Japan). To obtain the time-lapse images, the cells (1 × 10^3^, 0.1 ml) were seeded in a clear-bottom 96-well black plate (Thermo Fisher Scientific). To visualize the S/G2/M phases, green fluorescent protein mAG (Ex/Em = 492 nm/505 nm), and the G1 phase, red fluorescent protein mKO2 (Ex/Em = 551 nm/565 nm), a fluorescence microscope BZ-X810 (Keyence Corporation) equipped with a Stage Top Incubator (Tokai HIT) was utilized.

### RNA extraction, purification, and reverse transcription quantitative PCR (RT-qPCR)

Total RNA was extracted from HeLa cells using NucleoSpin RNA Plus (Takara Bio), and the contaminated DM in RNA samples was removed using OneStep PCR Inhibitor Removal Kits (Zymo Research) according to the manufacturer’s instructions. The highly purified RNA was reverse-transcribed to cDNA using ReverTraAce (TOYOBO), and qPCR analysis was performed using the THUNDERBIRD SYBR-Green real-time PCR system (TOYOBO) and CFX96 Touch Real-Time PCR detection system (Bio-Rad). The primers used are described in Table S1.

### Gene silencing by RNA interference

For transient knockdown experiments, siRNA was used according to the manufacturer’s instructions. Briefly, for AMBRA1 and GSK-3β knockdown, HeLa cells (1.5 × 10^5^, 3 ml) were seeded in 6-well plates and cultured overnight. Then, 30 pmol of siRNA was mixed with 9 μl of Lipofectamine RNAiMAX reagent (Invitrogen) in 300 μl of Opti-MEM I Reduced Serum Medium (Invitrogen), and 250 μl of the mixture was used for transfection (forward transfection method). For cyclin D1 and cyclin D3 knockdown performed in 96-well plates, 5 pmol of siRNA were mixed with 1.5 μl of Lipofectamine RNAiMAX reagent in 50 μl of Opti-MEM I Reduced Serum Medium, and 10 μl of this mixture was added to each well. Subsequently, HeLa cells (5 × 10^3^cells in 100 μl) were added to the wells (reverse transfection method). MISSION siRNA Universal Negative Control siRNA #1 (for control), MISSION siRNA SASI_Hs01_00116731 and SASI_Hs01_00116732 (for AMBRA1), MISSION siRNA SASI_Hs01_00192106 and SASI_Hs01_00192104 (for GSK-3β), MISSION siRNA SASI_Hs01_00213909 (for cyclin D1), and MISSION siRNA SASI_Hs01_00050184 (for cyclin D3) were purchased from Sigma-Aldrich. The siRNAs used are described in Table S3.

### Mice and allograft transplantation

Eight-week-old female SPF Balb/c nude mice (n = 10) with body weights ranging from 17 to 22 g were purchased from Japan SLC, Inc., and maintained in a humid environment (50 ± 10%) under a 12-h light/dark cycle in a temperature-controlled (22 ± 2 °C) pathogen-free animal facility. Three mice were housed in each cage; the mice were fed a standard chow diet (CRF-1; Oriental Yeast Co., Ltd, Tokyo, Japan) and provided with tap water filtered by a polypropylene string wound cartridge (Organo Co., Ltd, Tokyo, Japan). The mice were allowed to acclimate for 1 week before the experiments. The B16F10 cells were subcutaneously injected into the dorsal flank of nude mice.

The Animal Care and Use Committee at Chubu University approved this study (approval no. 3010039 and 202010016).

### Immunofluorescence

Tumor masses were excised and fixed overnight in 10% neutral buffered formalin. The paraffin-embedded tissues were sectioned at 4 μm, deparaffinized in xylene, and rehydrated through a graded ethanol series. Antigen retrieval was performed using epitope retrieval solution pH 9 (RE7119-CE, Leica Biosystems) at 98 °C for 30 min. After cooling to room temperature, the sections were incubated with blocking solution (5% normal goat serum in PBS) for 60 min. Sections were then incubated overnight at 4 °C with primary antibodies against Ki-67 or cyclin D1, followed by incubation with Alexa Fluor 647-conjugated goat anti-rabbit IgG secondary antibody (Funakoshi) for 30 min at room temperature. Nuclei were counterstained with DAPI. Fluorescence images were acquired and analyzed using ImageJ software. Ki-67- or cyclin D1-positive cells were identified based on fluorescence intensity above a predefined threshold, and positivity was expressed as the percentage of marker-positive nuclei relative to the total number of DAPI-positive nuclei.

### Statistical analysis

Statistical analyses were performed using Statcel 4 (OMS Publishing), add-in software for Microsoft Excel, and R version 4.4.1 (R Foundation for Statistical Computing) (40). Normality was assessed using the Shapiro–Wilk test for *in vitro* experimental data with three or more replicates. Parametric tests were performed for normally distributed data, while non-parametric tests were applied to non-normally distributed data. For comparisons between multiple groups, a one-way analysis of variance (ANOVA) was conducted. When ANOVA showed significant differences, the Tukey–Kramer test and Dunnett’s test were performed as *post hoc* tests. For comparisons between the two groups, the Mann–Whitney U test was performed for non-normally distributed data. For non-parametric analysis of multiple groups, Kruskal–Wallis test was conducted, followed by Dunn’s *post hoc* test with Bonferroni correction. Statistical significance was defined as ∗*p*< 0.05 and ∗∗*p*< 0.01.

## Data availability

All data are available in the main text, figures, and supporting information.

## Supporting information

This article contains supporting information.

## Conflict of interest

The authors declare that they do not have any conflicts of interest with the content of this article.

## Supporting information

Figure S6

Figure S2A

Figure S2B
