# Supplementary material for: A novel synthetic melanin as a potential anticancer agent that induces apoptosis and cyclin D downregulation through distinct pathways
Source: J Biol Chem. 2026 Apr 24;302(6):113065. doi: 10.1016/j.jbc.2026.113065 (PMC13197775; doi:10.1016/j.jbc.2026.113065)
Supplement: Table S1 [file mmc1.docx]

Table S1

List of primers used in RT-qPCR

Gene name

Cyclin D1

Cyclin D1

Cyclin D3

Cyclin D3

GAPDH

GAPDH

Sequence (5′→3′)

TATTGCGCTGCTACCGTTGA

CCAATAGCAGCAAACAATGTGAAA

CCTCAATCTGCTCCTGGCAA

ATTCTGCACCGGCTCTCTC

ACAACTTTGGTATCGTGGAAGG

GCCATCACGCCACAGTTTC

Forward

Reverse

Primer

Forward

Reverse

Forward

Reverse
