# Supplementary material for: A novel synthetic melanin as a potential anticancer agent that induces apoptosis and cyclin D downregulation through distinct pathways
Source: J Biol Chem. 2026 Apr 24;302(6):113065. doi: 10.1016/j.jbc.2026.113065 (PMC13197775; doi:10.1016/j.jbc.2026.113065)
Supplement: Table S2 [file mmc2.docx]

Table S2

List of specific antibodies used for Western blotting and Immunohistochemistry

| 1^st^ Antibody | Source | Product number | 2^nd^ Antibody | Label | Source | Product number |
| --- | --- | --- | --- | --- | --- | --- |
| Cyclin A2 | CST | #4656 | Anti-mouse IgG | HRP | CST | #7076 |
| Cyclin B1 | CST | #4138 | Anti-rabbit IgG | HRP | CST | #7074 |
| Cyclin D1 | CST | #2978 | Anti-rabbit IgG | HRP | CST | #7074 |
| Cyclin D2 | CST | #3741 | Anti-rabbit IgG | HRP | CST | #7074 |
| Cyclin D3 | CST | #2936 | Anti-mouse IgG | HRP | CST | #7076 |
| Cyclin E1 | CST | #4129 | Anti-mouse IgG | HRP | Dako | #P0447 |
| Cyclin E2 | CST | #4132 | Anti-rabbit IgG | HRP | CST | #7074 |
| Cyclin H | CST | #2927 | Anti-rabbit IgG | HRP | CST | #7074 |
| CDK1/CDC2 | CST | #9112 | Anti-rabbit IgG | HRP | CST | #7074 |
| CDK2 | CST | #2546 | Anti-rabbit IgG | HRP | CST | #7074 |
| CDK4 | CST | #2906 | Anti-mouse IgG | HRP | CST | #7076 |
| CDK6 | CST | #3136 | Anti-mouse IgG | HRP | CST | #7076 |
| p15 INK4b | CST | #4822 | Anti-rabbit IgG | HRP | CST | #7074 |
| p57 Kip2 | Proteintech | #66794-1-Ig | Anti-mouse IgG | HRP | CST | #7076 |
| β-Tubulin | CST | #2128 | Anti-rabbit IgG | HRP | CST | #7074 |
| PARP | CST | #9542 | Anti-rabbit IgG | HRP | CST | #7074 |
| Cleaved PARP | CST | #5625 | Anti-rabbit IgG | HRP | CST | #7074 |
| Caspase-3 | CST | #9662 | Anti-rabbit IgG | HRP | CST | #7074 |
| Cleaved Caspase-3 | CST | #9661 | Anti-rabbit IgG | HRP | CST | #7074 |
| Cleaved Caspase-7 | CST | #9492 | Anti-rabbit IgG | HRP | CST | #7074 |
| GSK-3β | Proteintech | #51065-1-AP | Anti-rabbit IgG | HRP | CST | #7074 |
| β-Catenin | Proteintech | #177565-I-AP | Anti-rabbit IgG | HRP | CST | #7074 |
| β-actin-HRP | MBL | #PM053-7 | - |  | - | - |
| Ki-67 | Abcam | AB15580 | Anti-rabbit IgG | Alexa Fluor 647 | Thermo | A-21245 |
| Cyclin D1 | CST | #55506 | Anti-rabbit IgG | Alexa Fluor 647 | Thermo | A-21245 |

CST: Cell Signaling Technology, MA, USA; Proteintech: Proteintech Group, IL, USA; Dako: Agilent Technologies (Dako), CA, USA; MBL: Medical & Biological Laboratories, Tokyo, Japan; Abcam: Abcam plc, Cambridge, UK; Thermo: Thermo Fisher Scientific, MA, USA

CST: Cell Signaling Technology, Inc, MA, USA

Proteintech: Proteintech Group, Inc, IL, USA

Dako: Agilent Technologies (Dako), CA, USA

MBL: MEDICAL ＆ BIOLOGICAL LABORATORIES CO., LTD., Japan

The specificity of all antibodies used in this study was confirmed according to the manufacturer's validation data.
