# Supplementary material for: A novel synthetic melanin as a potential anticancer agent that induces apoptosis and cyclin D downregulation through distinct pathways
Source: J Biol Chem. 2026 Apr 24;302(6):113065. doi: 10.1016/j.jbc.2026.113065 (PMC13197775; doi:10.1016/j.jbc.2026.113065)
Supplement: Table S3 [file mmc3.docx]

Table S3

List of siRNA sequences used in this study

Sequence (5'→3')

Target

Sense: CCACAGAUGUGAAGUUCAU[dT][dT]

Antisense: AUGAACUUCACAUCUGUGG[dT][dT]

Cyclin D1

Sense: CAGAUUAUACCUUUGCCAU[dT][dT]

Antisense: AUGGCAAAGGUAUAAUCUG[dT][dT]

Cyclin D3

Sense: GGACUAUGUUCCGGAAACA[dT][dT]

Antisense: UGUUUCCGGAACAUAGUCC[dT][dT]

GSK-3β (#1)

Sense: GACACUAUAGUCGAGCCAA[dT][dT]

Antisense: UUGGCUCGACUAUAGUGUC[dT][dT]

GSK-3β (#2)

Sense: CAUUGAAUCUGAGAGGCCA[dT][dT]

Antisense: UGGCCUCUCAGAUUCAAUG[dT][dT]

AMBRA1(#1)

Sense: GCAUUCACCGCAGCUCUCA[dT][dT]

Antisense: UGAGAGCUGCGGUGAAUGC[dT][dT]

AMBRA1(#2)

Proprietary sequence

Negative control
