# Supplementary material for: A novel synthetic melanin as a potential anticancer agent that induces apoptosis and cyclin D downregulation through distinct pathways
Source: J Biol Chem. 2026 Apr 24;302(6):113065. doi: 10.1016/j.jbc.2026.113065 (PMC13197775; doi:10.1016/j.jbc.2026.113065)
Supplement: Figure S1 [file mmc4.pdf]

Figure S1

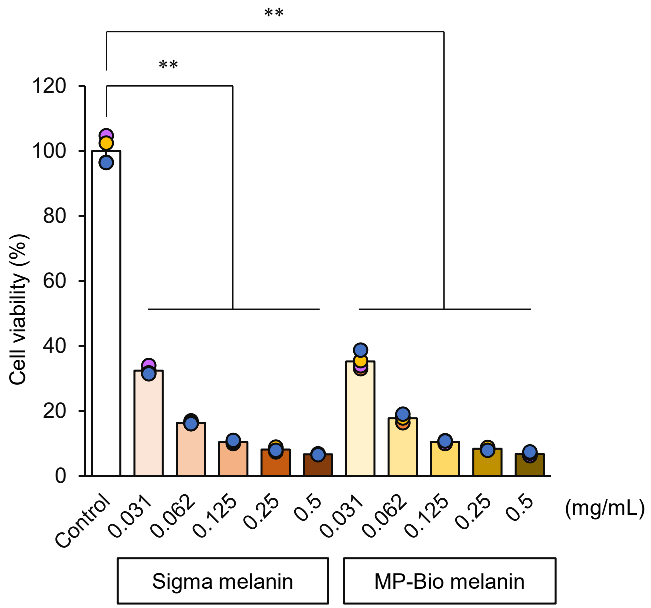

Growth inhibition of HeLa cells by commercially available melanins processed by sonication. Melanin powders obtained from Sigma and MP Biomedicals were suspended in distilled water at a final concentration of 50 mg/mL and subjected to ultrasonic disruption using a Bioruptor 2 (Sonic Bio Co., Ltd., Tokyo, Japan) with 30 s intervals for a total of 30 min. HeLa cells were seeded into 96-well plates, and various concentrations of the sonicated melanin suspensions were added. After 42 h of incubation, the plates were washed, and cell viability was determined using a WST-8 assay. The graph shows the relative cell viability compared with the untreated control. Data are presented as mean ± SD (n = 4 per group). Statistical significance was assessed using one-way ANOVA, followed by Dunnett's *post hoc* test comparing each treatment group to the control. Sigma melanin: $F(5, 18) = 1593$, $p < 2 \times 10^{-16}$; MP-Bio melanin: $F(5, 18) = 1216$, $p < 2 \times 10^{-16}$. **$p < 0.01$.
