# Supplementary material for: A novel synthetic melanin as a potential anticancer agent that induces apoptosis and cyclin D downregulation through distinct pathways
Source: J Biol Chem. 2026 Apr 24;302(6):113065. doi: 10.1016/j.jbc.2026.113065 (PMC13197775; doi:10.1016/j.jbc.2026.113065)
Supplement: Figure S2 [file mmc5.docx]

Figure S2

Time-lapse morphological observation of DM-treated cultured cells. Time-lapse imaging was performed using a fluorescence microscope equipped with an incubator. *A*. HeLa/Fucci cells were treated with 0.125 mg/mL or 0.5 mg/mL DM and cultured for 96 h. *B*. HaCaT cells, WI-38 cells, and HeLa/Fucci cells were treated with 0.25 mg/mL DM and cultured for 72 h. An equal volume of water was added to the control cells. Fluorescence and bright-field images were captured at 1.5-hour intervals. The resulting images were then composited to create time-lapse videos. For HaCaT and WI-38 cells, which do not express fluorescent proteins, only bright-field images are shown. The videos presented are representative of the observed data.
