# Supplementary material for: A novel synthetic melanin as a potential anticancer agent that induces apoptosis and cyclin D downregulation through distinct pathways
Source: J Biol Chem. 2026 Apr 24;302(6):113065. doi: 10.1016/j.jbc.2026.113065 (PMC13197775; doi:10.1016/j.jbc.2026.113065)
Supplement: Figure S3 [file mmc6.pdf]

Figure S3

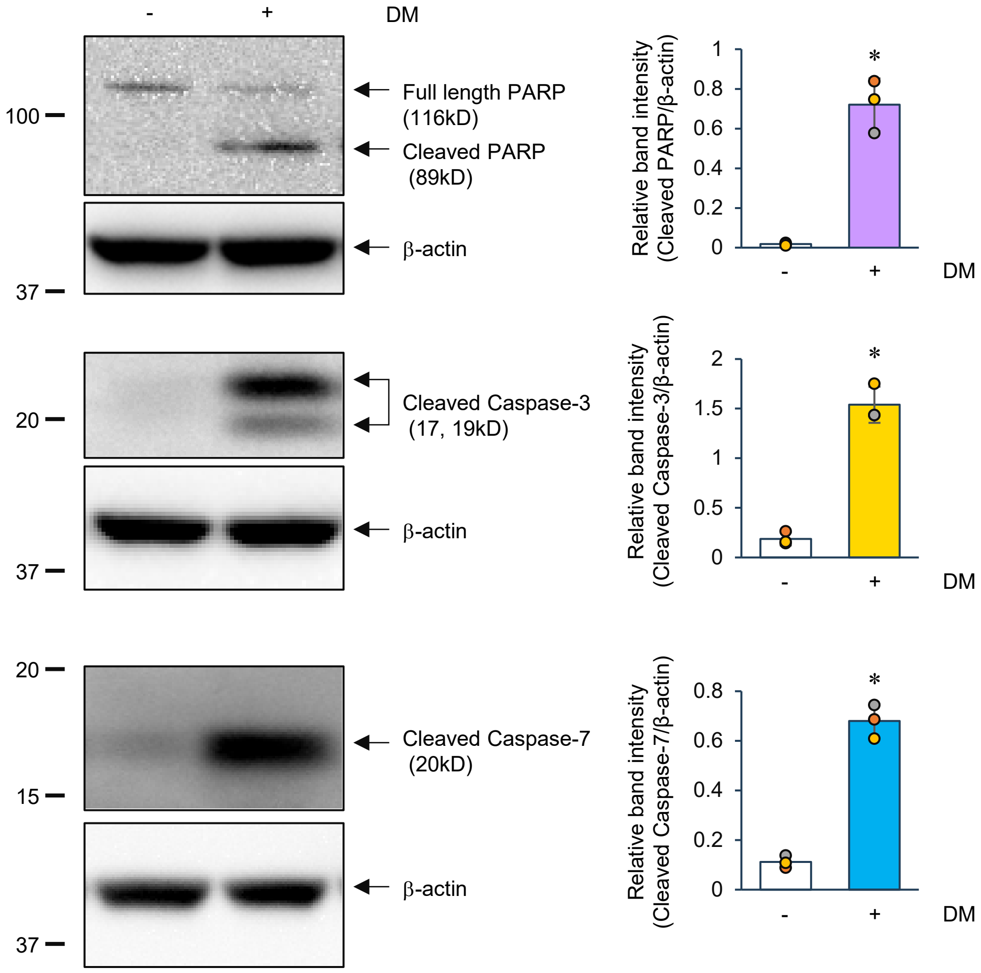

Examination of caspase involvement in DM-induced cell death. HeLa cells were treated with DM (0.25 mg/mL), and after 24 h, cells were harvested and soluble proteins were extracted. Western blotting was performed using specific antibodies to detect the indicated target proteins. PARP was detected as a full-length form (116 kDa) and a cleaved form (89 kDa). Cleaved caspase-3 (17 and 19 kDa) and cleaved caspase-7 (20 kDa) were also detected. Representative Western blot images are shown (left panel). β-actin was used as a loading control. Band intensities were quantified using ImageJ software, as described in the Experimental procedures section, normalized to β-actin, and expressed as relative band intensities of the target proteins. Quantified data are shown as graphs (right panel).
