# Supplementary material for: A novel synthetic melanin as a potential anticancer agent that induces apoptosis and cyclin D downregulation through distinct pathways
Source: J Biol Chem. 2026 Apr 24;302(6):113065. doi: 10.1016/j.jbc.2026.113065 (PMC13197775; doi:10.1016/j.jbc.2026.113065)
Supplement: Figure S4 [file mmc7.pdf]

Figure S4

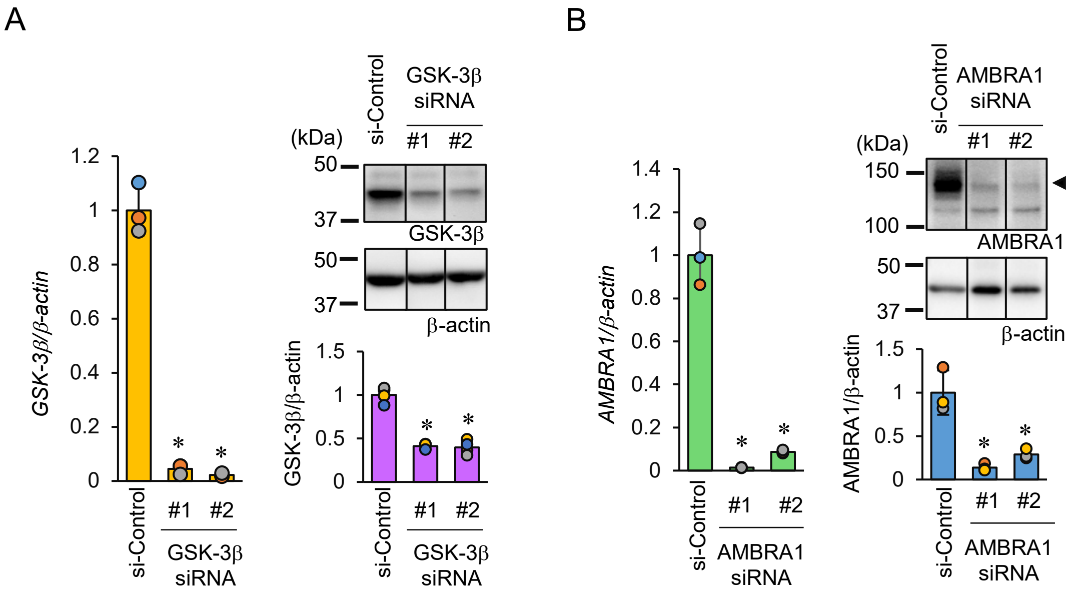

Validation of GSK-3β and AMBRA1 knockdown. HeLa cells were transfected with two independent siRNAs targeting GSK-3β (*A*) or AMBRA1 (*B*), and knockdown efficiency was verified by RT-qPCR (left panel) and Western blotting (right panel). Relative mRNA expression levels and band intensities of protein signals were quantified using ImageJ software, as described in the Experimental procedures section, and normalized to control siRNA-transfected cells. Statistical analysis was performed using three independent biological replicates with a two-tailed Mann–Whitney U test. Statistical significance was determined relative to control siRNA-transfected cells (*$p < 0.05$). β-actin was used as a loading control.
