# Supplementary material for: A novel synthetic melanin as a potential anticancer agent that induces apoptosis and cyclin D downregulation through distinct pathways
Source: J Biol Chem. 2026 Apr 24;302(6):113065. doi: 10.1016/j.jbc.2026.113065 (PMC13197775; doi:10.1016/j.jbc.2026.113065)
Supplement: Figure S5 [file mmc8.pdf]

Figure S5

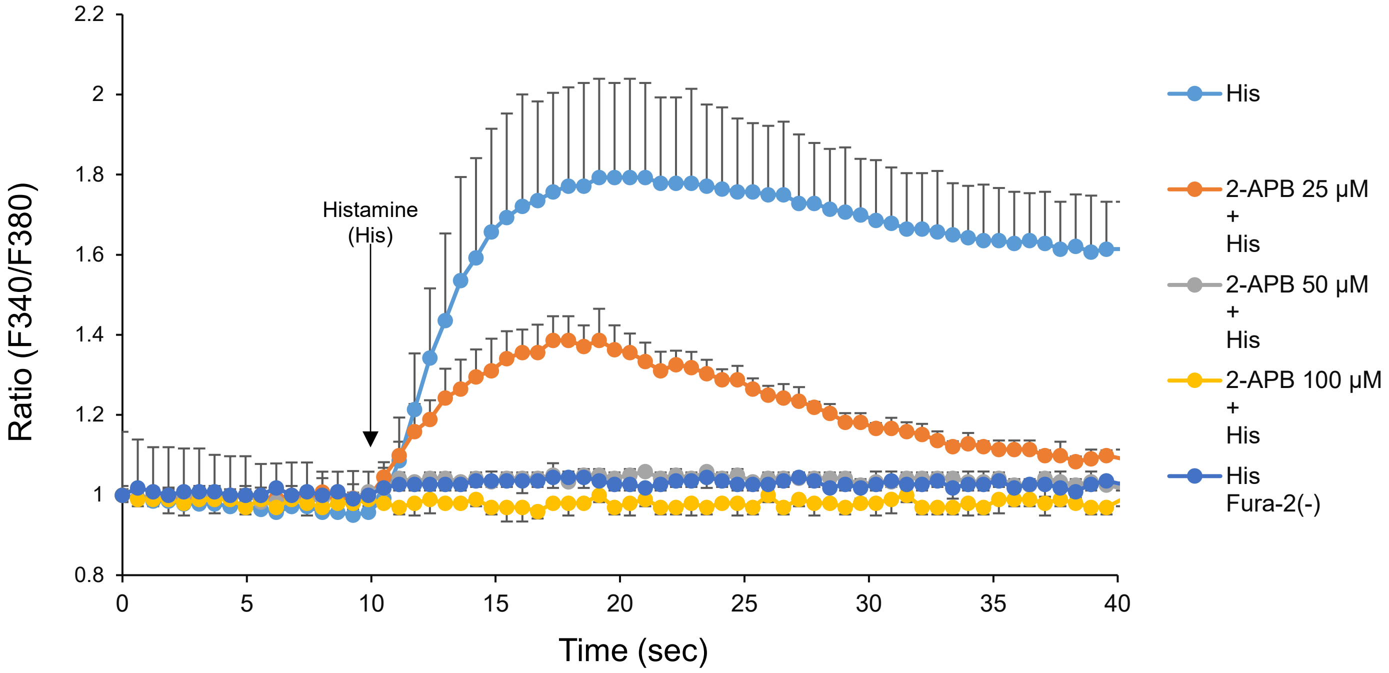

Detection of histamine-induced intracellular calcium and determination of the inhibitory concentration of 2-APB. HeLa cells (2×10^4) were seeded in clear-bottom 96-well black plates and pretreated with 2-APB (25, 50, or 100 μM) for 30 min. Cells were then incubated at 37° C in a CO_2 incubator with loading buffer (5 μM Fura-2, 0.04% Pluronic F-127, 1.25 mM probenecid) for 1 h, followed by replacement with recording medium (1.25 mM probenecid) prewarmed to 37° C. Fluorescence was measured with a spectrofluorometer at excitation/emission of 380/510 nm and 340/510 nm for 10 s, after which histamine (50 μM) was added and fluorescence kinetics were recorded for 30 s. The ratio of fluorescence intensities (F340/F380) was calculated, and relative values normalized to the baseline were plotted. Data represent n = 3 per group.
