# Supplementary material for: A novel synthetic melanin as a potential anticancer agent that induces apoptosis and cyclin D downregulation through distinct pathways
Source: J Biol Chem. 2026 Apr 24;302(6):113065. doi: 10.1016/j.jbc.2026.113065 (PMC13197775; doi:10.1016/j.jbc.2026.113065)
Supplement: Figure S6 [file mmc9.pdf]

Figure S6

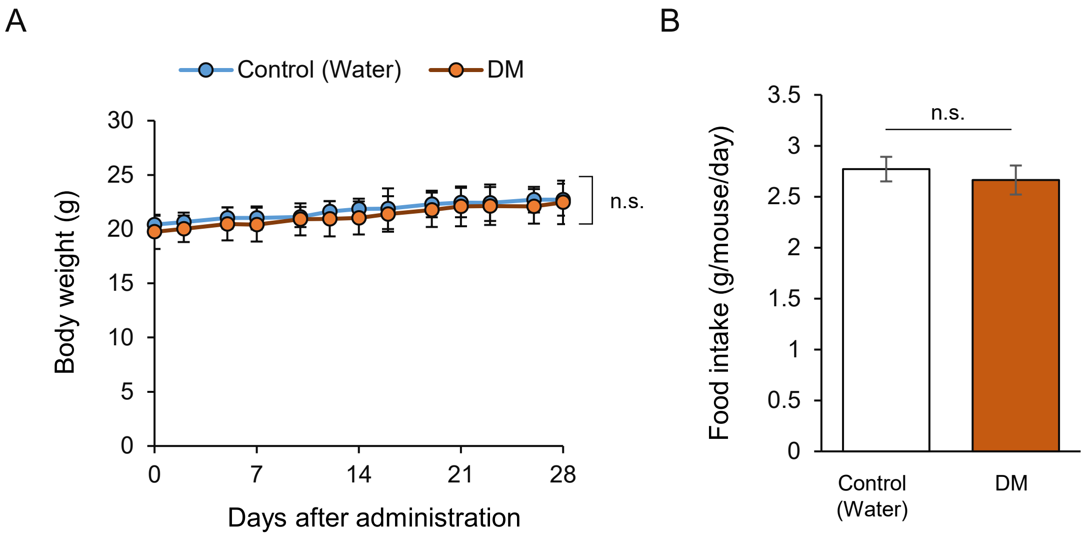

Effects of oral DM administration on body weight and food intake in mice. Six-week-old male C57BL/6 mice (Japan SLC, Inc., Japan) were administered DM (100 mg/mL, 0.2 mL/mouse, 1000 mg/kg body weight) or vehicle (distilled water, 0.2 mL/mouse) by oral gavage every 2–3 days for 4 weeks. Mice were fed AIN-93M diet ad libitum throughout the experiment. Data are expressed as mean ± SD (n = 6/group). (A) Body weight was measured every few days. No significant differences were detected between the Control and DM groups at any time point (two-way repeated-measures ANOVA; group effect: $F(1, 10) = 0.427$, $p = 0.528$; group × day interaction: $F(12, 120) = 0.541$, $p = 0.884$; Bonferroni post hoc test). (B) Mean daily food intake over the 4-week period is shown as a bar graph. No significant difference was observed between the groups (control: 2.77 ± 0.12 g/mouse/day; DM: 2.66 ± 0.14 g/mouse/day; Welch's t-test, n.s.).
